# Investigation of Maternal Effects, Maternal-Fetal Interactions and Parent-of-Origin Effects (Imprinting), Using Mothers and Their Offspring

**DOI:** 10.1002/gepi.20547

**Published:** 2011-01

**Authors:** Holly F Ainsworth, Jennifer Unwin, Deborah L Jamison, Heather J Cordell

**Affiliations:** School of Mathematics and Statistics, Newcastle UniversityNewcastle upon Tyne, United Kingdom; Institute of Human Genetics, Newcastle UniversityNewcastle upon Tyne, United Kingdom

**Keywords:** epigenetic, log-linear model, case/parent trio

## Abstract

Many complex genetic effects, including epigenetic effects, may be expected to operate via mechanisms in the inter-uterine environment. A popular design for the investigation of such effects, including effects of parent-of-origin (imprinting), maternal genotype, and maternal-fetal genotype interactions, is to collect DNA from affected offspring and their mothers (case/mother duos) and to compare with an appropriate control sample. An alternative design uses data from cases and both parents (case/parent trios) but does not require controls. In this study, we describe a novel implementation of a multinomial modeling approach that allows the estimation of such genetic effects using either case/mother duos or case/parent trios. We investigate the performance of our approach using computer simulations and explore the sample sizes and data structures required to provide high power for detection of effects and accurate estimation of the relative risks conferred. Through the incorporation of additional assumptions (such as Hardy-Weinberg equilibrium, random mating and known allele frequencies) and/or the incorporation of additional types of control sample (such as unrelated controls, controls and their mothers, or both parents of controls), we show that the (relative risk) parameters of interest are identifiable and well estimated. Nevertheless, parameter interpretation can be complex, as we illustrate by demonstrating the mathematical equivalence between various different parameterizations. Our approach scales up easily to allow the analysis of large-scale genome-wide association data, provided both mothers and affected offspring have been genotyped at all variants of interest. *Genet. Epidemiol.* 35:19–45, 2011. © 2010 Wiley-Liss, Inc.

## INTRODUCTION

The current era of genome-wide association studies has popularized the case/control design for the detection of genetic variants predisposing to complex diseases. However, as recently pointed out by Buyske [2008], associations detected in a case/control study can arise not only from genetic effects operating in the cases but also from alternative mechanisms that are statistically confounded with case genotype effects, such as maternal genotype effects, maternal-fetal interactions, or parent-of-origin (imprinting) effects. A variety of diseases, particularly those related to pregnancy outcomes or complications in utero, have been hypothesized to operate via such mechanisms. For example, both maternal and fetal genes, either individually or in combination, have been implicated in risk of pre-eclampsia [Goddard et al., 2007; Schneider et al., 1994; Wilson et al., 2003], low birthweight [Larizza et al., 2005; Ober et al., 1987], spina bifida [Jensen et al., 2006], and schizophrenia [Palmer et al., 2006]. With data collected only on cases and controls, these different types of effect will be indistinguishable. For example, a strong maternal genotype effect may present the same pattern of risks as a weak offspring (case) genotype effect, since cases and mothers of cases share an allele in common. Given family rather than case/control data, however—specifically given genotype data for cases plus their mothers and/or fathers—it may be possible to distinguish between these different mechanisms [Cordell et al., 2004; Hsieh et al., 2006; Shi et al., 2008; Sinsheimer et al., 2003; Weinberg, 1999b; Weinberg et al., 1998; Weinberg and Shi, 2009].

A popular design for the investigation of maternal effects and maternal-fetal interactions (operating perhaps via the inter-uterine environment) is to collect DNA from offspring and their mothers [Shi et al., 2008]. A comparison of the genotype relative risks in cases (displaying some disease of interest) vs. controls compared to the relative risks in mothers of cases vs. mothers of controls can allow one to investigate the merits of different competing underlying models. For example, Jamieson et al. [2008] found unusual patterns of risk when analyzing children affected with clinical signs of congenital toxoplasmosis vs. controls compared to when analyzing mothers of affected children vs. mothers of controls, a result that they interpreted as indicating the presence of a maternal genotype and/or imprinting effect.

More formally, given genotype data from “duos” consisting of offspring (affected or unaffected) together with their mothers, one may fit models (via logistic regression, for example) that incorporate effects of offspring genotype, maternal genotype, maternal-fetal interactions, and imprinting [Chen et al., 2009; Li et al., 2009; Shi et al., 2008; Weinberg and Umbach, 2005]. By examining the fit of the model with and without the inclusion of specific terms, one may test formally for their significance and estimate the magnitude of their effects. However, collinearities between the parameters representing the various effects can complicate the interpretation of such an analysis, as we shall discuss in more detail later.

A number of authors have considered the alternative approach of using case/parent trios (i.e. affected offspring and their parents) for the estimation of such effects [Chen et al., 2009; Cordell et al., 2004; Hsieh et al., 2006; Sinsheimer et al., 2003; Weinberg, 1999b; Weinberg et al., 1998]. Case/parent trios are often used in genetic association studies because of the robustness to population stratification they can provide, via use of family-based tests that examine the transmission of high-risk alleles from parents to affected offspring [Spielman et al., 1993]. Recently, however, the case/control design has achieved greater popularity owing to the larger sample size (and thus greater power) that is achievable [WTCCC, 2007] and the development of alternative methods to deal with population stratification [Devlin and Roeder, 1999; Price et al., 2006; Pritchard et al., 2000]. With case/parent trios, we can test for association and estimate genotype and haplotype relative risks using conditional logistic regression [Cordell and Clayton, 2002; Schaid, 1996; Schaid and Sommer, 1993] or log-linear modeling [Shi et al., 2009]. More complex effects, such as maternal genotype effects, maternal-fetal interactions and parent-of-origin effects, may be estimated through an extension of the conditional logistic regression approach [Cordell et al., 2004] or through log-linear modeling [Sinsheimer et al., 2003; Vermeulen et al., 2009; Weinberg, 1999b; Weinberg et al., 1998]. One of the merits of the case/parent trio design is the fact that it does not require control data: essentially the untransmitted parental alleles or genotypes are used as “controls” for the transmitted alleles or genotypes. However, greater efficiency can potentially be achieved by incorporation of one or more additional separate control samples consisting either of unrelated controls [Epstein et al., 2005; Nagelkerke et al., 2000], the parents of unrelated controls [Weinberg and Umbach, 2005], or of control/mother duos [Vermeulen et al., 2009]. Regardless of whether or not such additional control samples are used, most approaches generally assume that both parents (the mother and the father) of cases are available in at least a subset of families [Weinberg, 1999a]. Shi et al. [2008], however, extended their log-linear modeling approach to apply to case/mother duos (for which no fathers' genotypes are available), on the assumption that, in common with logistic regression, there exists a sample of control/mother duos that can be incorporated into the analysis. Given such a sample, Chen et al. [2009] developed an alternative constrained retrospective likelihood approach that exploits the Mendelian correlation between mother's and child's genomes under a Hardy-Weinberg equilibrium (HWE) assumption, allowing the estimation of maternal and child effects and their interactions, where the effects in the child and mother could operate either at the same locus or at different (separate) loci that are in linkage disequilibrium.

## METHODS

### NOTATION, PARAMETERIZATION, AND RELATIONSHIP TO PREVIOUS MODELS

Before describing our approach in detail, we introduce some notation. Without loss of generality, we denote the allele (at a particular genetic locus) that is expected to confer high risk as 2 and the low-risk allele as 1. (If, in fact, it is allele 1 that confers higher risk, the genotype relative risk estimates associated with allele 2 will turn out to be less than, rather than greater than, 1.0.) We parameterize the risks as follows: α corresponds to the baseline probability of disease for an individual (child) with the low-risk homozygote genotype (i.e. 11) whose parents are also homozygous 11. The parameters *R*_1_ and *R*_2_ correspond to multiplicative factors by which the child's probability of disease is multiplied if the child has one or two copies of the high-risk allele (i.e. has genotype 12 or 22, respectively). *S*_1_ and *S*_2_ correspond to multiplicative factors by which the child's probability of disease is multiplied if their mother has one or two copies of the high-risk allele, respectively. The parameters γ_11_, γ_12_, γ_21_, and γ_22_ are standard statistical interaction terms for the interaction between mother's and child's genotype (i.e. γ_*ij*_ is the (additional) factor by which the disease risk is multiplied when the mother has *i* copies and the child has *j* copies of the high-risk allele). The imprinting parameter *I*_m_ corresponds to a multiplicative factor by which the probability of disease is multiplied if the child receives a (maternal) copy of the high-risk allele from their mother, and the imprinting parameter *I*_p_ corresponds to a multiplicative factor by which the probability of disease is multiplied if the child receives a (paternal) copy of the high-risk allele from their father. Note that this notation for the parent-of-origin effects *I*_m_ and *I*_p_ corresponds to notation used by Weinberg et al. [1998] rather than to a later alternative parameterization used by Weinberg [1999b] (discussed further later). Notation for the other effects corresponds to the notation used by Weinberg et al. [1998] and Cordell et al. [2004], except for the parameters γ_*ij*_, which were not considered by Weinberg et al. [1998] or Weinberg [1999b] and which were previously denoted α_*ij*_ by Cordell et al. [2004].

The rationale for the above parameterization and its relationship to some alternative proposed parameterizations can be seen by examination of Table I. The quantitities given in each cell of Table I correspond to the probability (or odds) of the child developing disease given the genotype combination (*g*_m_, *g*_c_) for mother and child, as might be estimated in a logistic regression analysis of case/mother duos vs. control/mother duos. Since only seven genotype combinations are allowable under Mendelian inheritance, a maximum of seven parameters will be estimable (in the absence of any additional information e.g. concerning father's genotype).

**TABLE I:** Parameterization of penetrances (if controls are of unknown disease status) or odds (if controls are unaffected) for logistic regression models using mothers and their offspring

			*g*_c_		
			
Example	Description	*g*_m_	11	12	22
1	Offspring genotype effects (relative to *g*_c_ = 11)	11	α	α*R*_1_	–
		12	α	α*R*_1_	α*R*_2_
		22	–	α*R*_1_	α*R*_2_
		
2	Maternal genotype effects (relative to *g*_m_ = 11)	11	α	α	–
		12	α*S*_1_	α*S*_1_	α*S*_1_
		22	–	α*S*_2_	α*S*_2_
		
3	Offspring and maternal genotype effects relative to (*g*_c_, *g*_m_) = (11, 11)	11	α	α*R*_1_	–
		12	α*S*_1_	α*R*_1_*S*_1_	α*R*_2_*S*_1_
		22	–	α*R*_1_*S*_2_	α*R*_2_*S*_2_
		
4	Offspring and maternal genotype effects relative to (*g*_c_, *g*_m_) = (22, 22)	11	α*R*_2_*S*_2_	α*R*_1_*S*_2_	–
		12	α*R*_2_*S*_1_	α*R*_1_*S*_1_	α*S*_1_
		22	–	α*R*_1_	α
		
5	Example of offspring and maternal genotype effects	11	0.05	0.10	–
		12	0.15	0.30	0.60
		22	–	0.40	0.80
		
6	Offspring and maternal genotype effects plus interactions	11	α	α*R*_1_	–
		12	α*S*_1_	α*R*_1_*S*_1_γ_11_	α*R*_2_*S*_1_γ_12_
		22	–	α*R*_1_*S*_2_γ_21_	α*R*_2_*S*_2_γ_22_
		
7	Saturated model	11	δ_00_	δ_01_	–
		12	δ_10_	δ_11_	δ_12_
		22	–	δ_21_	δ_22_
		
8	Sinsheimer et al. [2003] parameterization 1A^a^	11	α	αρ_1_µ_0_	–
		12	αη_1_	αρ_1_η_1_	αρ_2_η_1_
		22	–	αρ_1_η_2_	αρ_2_η_2_
		
9	Sinsheimer et al. [2003] parameterization 1B	11	α	αρ_1_µ_0_	–
		12	αη_1_	αρ_1_η_1_	αρ_2_η_1_
		22	–	αρ_1_η_2_µ_2_	αρ_2_η_2_
		
10	Palmer et al. [2006] and Sinsheimer et al. [2003] parameterization 2	11	αµ	αρ_1_	–
		12	αη_1_µ	αρ_1_η_1_µ	αρ_2_η_1_µ
		22	–	αρ_1_η_2_	αρ_2_η_2_µ
		
11	Offspring and maternal effects plus imprinting [Weinberg et al., 1998]	11	α	α*R*_1_*I*_p_	–
		12	α*S*_1_	α*R*_1_*S*_1_ (*A*_1_*I*_m_+*A*_2_*I*_p_)	α*R*_2_*S*_1_*I*_m_*I*_p_
		22	–	α*R*_1_*S*_2_*I*_m_	α*R*_2_*S*_2_*I*_m_*I*_p_
		
12	Offspring+maternal effects plus maternal imprinting [Weinberg et al., 1998]	11	α	α*R*_1_	–
		12	α*S*_1_	α*R*_1_*S*_1_ (*A*_1_*I*_m_+*A*_2_)	α*R*_2_*S*_1_*I*_m_
		22	–	α*R*_1_*S*_2_*I*_m_	α*R*_2_*S*_2_*I*_m_
		
13	Offspring+maternal effects plus paternal imprinting [Weinberg et al., 1998]	11	α	α*R*_1_*I*_p_	–
		12	α*S*_1_	α*R*_1_*S*_1_ (*A*_1_+*A*_2_*I*_p_)	α*R*_2_*S*_1_*I*_p_
		22	–	α*R*_1_*S*_2_	α*R*_2_*S*_2_*I*_p_
		
14	Maternal imprinting effects only	11	α	α	–
		12	α	α(*A*_1_*I*_m_+*A*_2_)	α*I*_m_
		22	–	α*I*_m_	α*I*_m_
		
15	Paternal imprinting effects only	11	α	α*I*_p_	–
		12	α	α(*A*_1_+*A*_2_*I*_p_)	α*I*_p_
		22	–	α	α*I*_p_
		
16	Offspring effects plus maternal imprinting [Weinberg et al., 1998]	11	α	α*R*_1_	–
		12	α	α*R*_1_ (*A*_1_*I*_m_+*A*_2_)	α*R*_2_*I*_m_
		22	–	α*R*_1_*I*_m_	α*R*_2_*I*_m_
		
17	Offspring effects plus maternal imprinting [Weinberg, 1999b]	11	α	α*R*_1_	–
		12	α	α*R*_1_(*A*_1_*I*_m_+*A*_2_)	α*R*_2_
		22	–	α*R*_1_*I*_m_	α*R*_2_
		
18	Offspring effects plus imprinting (alternative parameterization)	11	α	α*R*_p_	–
		12	α	α(*A*_1_*R*_m_+*A*_2_*R*_p_)	α*R*_2_
		22	–	α*R*_m_	α*R*_2_
		
19	Offspring and maternal genotype effects plus interactions and imprinting	11	α	α*R*_1_*I*_p_	–
		12	α*S*_1_	α*R*_1_*S*_1_γ_11_(*A*_1_*I*_m_+*A*_2_*I*_p_)	α*R*_2_*S*_1_γ_12_*I*_m_*I*_p_
		22	–	α*R*_1_*S*_2_γ_21_*I*_m_	α*R*_2_*S*_2_γ_22_*I*_m_*I*_p_
		
20	Parimi et al. [2008] and Li et al. [2009] paramerization^b^	11	α	α*R*_1_*j*_m_*j*_c_	–
		12	α*S*_1_*j*_c_	α*R*_1_*S*_1_ (*A*_1_+*A*_2_*j*_m_)	α*R*_2_*S*_1_*j*_c_
		22	–	α*R*_1_*S*_2_*j*_c_	α*R*_2_*S*_2_

a The parameter denoted µ_0_ here was actually denoted µ by Sinsheimer et al. [2003].

b The parameters shown here correspond to the parameters in the notation of Table 1 of Li et al. [2009] as follows: *j*_m_ = exp(*i*_m_), *j*_m_ = exp(*i*_c_), *A*_1_ = π_1_, *A*_2_ = π_2_, α = exp(µ−*a*_m_−*a*_0_), *R*_1_ = exp(*a*_0_+*d*_0_), *R*_2_ = exp(2*a*_0_), *S*_1_ = exp(*a*_m_+*d*_m_), *S*_2_ = exp(2*a*_m_).

The first illustrative example (shown in the top three rows of Table I) shows a parameterization where child genotype effects only are assumed to operate. Relative to the baseline penetrance α, the penetrance is increased by a factor *R*_1_ if a child has one copy and by a factor *R*_2_ if a child has two copies of allele 2. The second illustrative example shows a parameterization where maternal effects only are assumed to operate: relative to the baseline penetrance α, the penetrance is increased by a factor *S*_1_ if the mother has one copy and by a factor *S*_2_ if the mother has two copies of allele 2. Example 3 in Table I shows a parameterization where both child and maternal genotypes operate. Example 4 shows a similar model except that in this case the maternal and child genotype effects are expressed relative to the baseline penetrance of the genotype combination (*g*_m_, *g*_c_) = (22, 22). Suppose the true penetrances are as shown in Example 5 of Table I. Then these penetrances could be precisely modeled using the parameterization of Example 3, with parameters (α, *R*_1_, *R*_2_, *S*_1_, and *S*_2_) taking the values (0.05, 2, 4, 3, and 4). Equally, these penetrances could be precisely modeled using the parameterization of Example 4, with parameters (α, *R*_1_, *R*_2_, *S*_1_, and *S*_2_) taking the values (0.8, 0.5, 0.25, 0.75, and 0.25). Either parameterization would provide an equally “correct” representation of the undelying penetrance structure and either would provide identical inference (when applied to observed data) concerning whether child genotype and/or maternal genotype effects exist. However, the values of the parameters (and thus the inference concerning whether these effects increase or decrease risk) depend on whether one is specifying the problem relative to the baseline genotype combination (*g*_m_, *g*_c_) = (11, 11) or to (*g*_m_, *g*_c_) = (22, 22).

Example 6 in Table I attempts to additionally include the four interaction effects (γ_11_, γ_12_, γ_21_, and γ_22_). This is a standard statistical parameterization of interaction effects defined as a departure from multiplicative effects of two factors on the relative risk or odds scale [Clayton, 2009; Cordell, 2009; Thomas, 2010]. Such a parameterization would generally lead to a saturated 9df model; however, in our example, since only seven categories are allowable under Mendelian inheritance, a 7df model is already saturated and not all nine parameters will be identifiable. We find that α, *R*_1_, *S*_1_, and γ_11_ are all identifiable. We can also identify three further “composite” parameters: *R*_2_γ_12_, *S*_2_γ_21_, and *R*_2_*S*_2_γ_22_ (or, equivalently, γ_22_/(γ_12_γ_21_)). If we are willing to assume that γ_12_ and γ_21_ are in fact equal to 1 (i.e. these two interation effects do not exist), then we can identify *R*_2_, *S*_2_, and γ_22_. However, our interpretation of the estimates of *R*_2_, *S*_2_, and γ_22_ we obtain depends crucially on this assumption. If our assumption is not correct, then the parameter we call *R*_2_ is, in fact, a composite product *R*_2_γ_12_ of child genotype and interaction effects, the parameter we call *S*_2_ is, in fact, a composite product *S*_2_γ_21_ of maternal genotype and interaction effects, and the parameter we call γ_22_ is a composite ratio γ_22_/(γ_12_γ_21_).

In order to avoid these complications of parameter interpretation, one might prefer to fit a 7df saturated model as shown in Example 7 of Table I. This fits a separate parameter δ_*ij*_ to each genotype combination (where the mother has *i* copies and the child *j* copies of allele 2). This model avoids the temptation to attribute any particular mechanism or meaning to the seven parameters estimated, but, in fact, we would quite like to be able to attribute meaning to the parameters estimated! In particular, we would like to be able to attribute effects as being due to either the mother's or the child's genotype (or both), as these possibilities lead to very different biological hypotheses concerning the underlying disease mechanism, and why it should be that the presence of certain alleles in the mother and/or child lead to an increase or decrease in disease risk. A further disadvantage of the 7df saturated model of Example 7 is the large number of df when comparing against the null hypothesis that all δ_*ij*_ are equal. A more natural approach might be to fit a sequence of nested models [see Fig. 3 of Cordell et al., 2004] where one starts by entering child and/or maternal effects before attempting to include interactions.

**Fig. 3:**
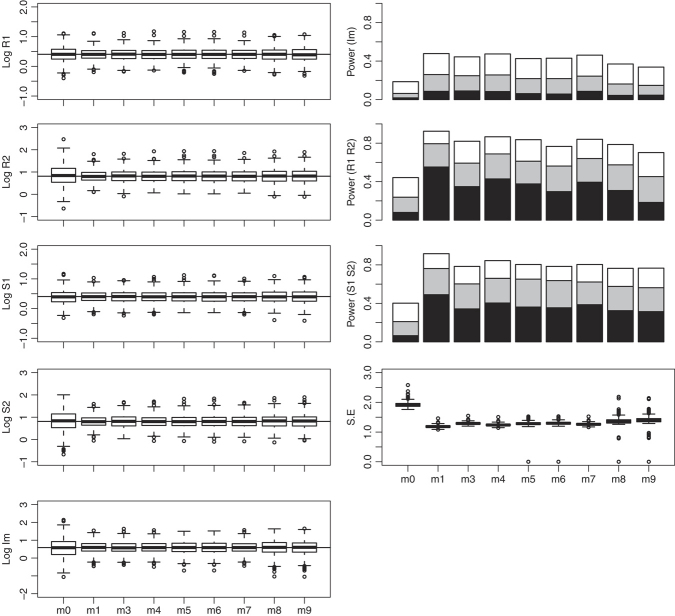
Results from simulation scenario F. See figure legend to Figure 1 for detailed description of plots. The different methods are denoted m_0_–m_9_. Method 2 is not shown as the allele frequency *A*_2_ was found to be unidentifiable using Method 2 when fitting scenarios F, G, H, I, and J. The left hand panels show boxplots of the relevant parameter estimates (logs of the given relative risk parameter) with a line indicating the true value, while the top three right panels show the power of likelihood ratio tests of various hypotheses (specifically of whether the given parameter(s) are zero, when allowing for the effects of the other parameters). The lowest right panel shows a boxplot of the total estimated standard error (SE) over the 500 simulation replicates.

The idea of choosing a parameterization that best represents an underlying biological reality was used by Sinsheimer et al. [2003]. These authors used the log-linear modeling framework of Weinberg et al. [1998] and Weinberg [1999b] to develop a test known as the maternal-fetal genotype incompatibility (MFG) test, a test that has recently been extended to apply to more general family structures than case/parent trios [Childs et al., 2010]. The parameterizations used by the two versions (Scenarios 1A and 1B) of the MFG test are shown as Examples 8 and 9 of Table I. The parameters (ρ_1_, ρ_2_, η_1_, and η_2_) of Sinsheimer et al. [2003] correspond closely in concept to our parameters (*R*_1_, *R*_2_, *S*_1_, and *S*_2_). Sinsheimer et al. [2003] included up to two interaction parameters which they denoted as µ (or µ_0_) and µ_2_ (slightly unfortunate notation in view of the fact that Weinberg [1999b] had already used the notation µ_1_−µ_6_ to refer to six mating-type stratification parameters, as described later). In our notation, these interaction parameters would probably best be denoted as γ_01_ and γ_21_, since they correspond to effects that operate (in addition to maternal and child genotype effects) when the child has one copy, and the mother has either zero or two copies, of a particular allele of interest. The rationale for including either one (µ_0_) or both (µ_0_ and µ_2_) of these parameters is that they encapsulate a biological mechanism whereby adverse effects (such as increased disease risks) can result from an incompatibility or “mismatch” between maternal and fetal genotypes, such as that which occurs in RhD-induced hemolytic disease [Strachan and Read, 1999].

The parameters used in Scenario 1B of Sinsheimer et al. [2003] can be shown to be expressible in terms of our (Example 6) parameters as follows: α = α, ρ_1_ = *R*_1_γ_11_, ρ_2_ = *R*_2_γ_12_, η_1_ = *S*_1_, η_2_ = *S*_2_γ_22_/γ_12_, µ_0_ = 1/γ_11_, µ_2_ = γ_21_γ_12_/(γ_11_γ_22_) (noting that, in practice, we would estimate only one of γ_21_, γ_12_, γ_22_, allowing us to similarly express our parameters in terms of those of Sinsheimer et al. [2003]). The likelihoods induced by the two parameterizations (ours and Sinsheimer's) under either the null hypothesis of no interaction effects or the alternative hypothesis (in which one or two interaction effects are estimated in addition to child and maternal genotype effects) are identical, and either parameterization will thus produce the same inference with respect to whether interaction effect(s) exist or not. However, the interpretation of the parameter estimates obtained will depend on the parameterization used. If, for example, our parameterization is “correct” and γ_12_ operates but no other interaction effects exist, then the parameters estimated under the MFG model will interpret γ_12_ as an induced incompatibility parameter µ_2_ = γ_12_, and the estimates of ρ_2_ and η_2_ will equal the true values of *R*_2_ and *S*_2_ multiplied by and divided by the true value of γ_12_, respectively. Similarly, if the Sinsheimer et al. [2003] parameterization is “correct,” then the parameters estimated under our model will be functions of these “true” parameter values. This point is illustrated in more detail later through analysis of a particular example data set (see Results).

Palmer et al. [2006] considered an alternative parameterization (also proposed as Scenario 2 of Sinsheimer et al. [2003]) in which “matching” rather “mismatching” between maternal and fetal genotypes increases disease risk in the offspring. This parameterization was designed for the multiallelic *HLA* system, but, when applied to a diallelic locus, leads to Example 10 of Table I. Comparison of this model to Example 9 (Scenario 1B of Sinsheimer et al. [2003]) shows that these models are equivalent if one makes the restriction µ_0_ = µ_2_, and writes the Palmer et al. [2006] parameter µ as µ = 1/µ_0_. As this model is more restricted than ours, it is complicated to write down the general relationship between our seven parameters and the six parameters of Palmer et al. [2006]; however, one possible equivalence occurs if we set γ_12_ = γ_21_ = γ_22_ = 1 and then set (α′,µ,ρ_2_,ρ_2_,η_1_,η_2_) (in Sinsheimer et al. [2003] parametrization) to equal (α/γ_11_,γ_11_,*R*_1_γ_11_,*R*_2_,*S*_1_,*S*_2_) in our parametrization.

Example 11 in Table I shows a parameterization proposed by Weinberg et al. [1998], in which no interactions operate but maternal (*I*_m_) and/or paternal (*I*_p_) imprinting effects are assumed to exist. By imprinting, we mean a phenomenon whereby the expression of an allele varies according to parental origin (maternal or paternal) of the allele [Wilkinson et al., 2007]. For example, at the gene encoding Insulin-like growth factor II, the only allele expressed is the one inherited from the father [DeChiara et al., 1991]. To capture such an effect (in addition to main effects, operating through other mechanisms, of the child's and mother's genotype), it seems reasonable to incorporate parameters that operate only when a child has received a copy of “2” allele from their mother and/or father, as shown in Example 11. The genotype combination where both mother and child are heterozygous does not allow the parental origin of the “2” allele in the child to be inferred, and so the overall penetrance in this cell is a weighted combination of the two underlying possibilities (one in which the child received the “2” allele from their mother, and one in which the child received the “2” allele from their father) [Li et al., 2009]. It can be shown (derivation not given) that, under Hardy Weinberg Equilibrium (HWE) and random mating, the relative weights for these two possibilities correspond to the allele frequencies (*A*_1_, *A*_2_ = 1−*A*_1_) of alleles “1” and “2,” respectively. Alternatively, given genotype data from fathers as well as mothers of affected/control individuals, it may be possible to infer parental origin, provided the father is not also heterozygous [Weinberg, 1999b].

Although the parameterization of Example 11 makes intuitive sense, examination of Table I shows that, regardless of whether parental origin is, or is not, inferrable, the parameter *R*_2_ always occurs in combination with the product *I*_m_*I*_p_, and *R*_1_ always occurs in combination with *I*_m_ or *I*_p_, meaning that we cannot estimate all four of these parameters: we can only estimate *R*_1_*I*_m_, *R*_1_*I*_p_, and *R*_2_*I*_m_*I*_p_. Altogether, therefore, we can identify six parameters: α, *S*_1_, *R*_1_*I*_p_, *R*_2_*I*_m_*I*_p_, *S*_2_, and *I*_m_/*I*_p_. With respect to pure imprinting, therefore, all we can estimate is the ratio of maternal to paternal gene expression *I*_m_/*I*_p_. If we are willing to assume that *I*_p_ equals 1, then we can estimate *I*_m_ (or vice versa) as well as estimating *R*_1_ and *R*_2_ (see Examples 12 and 13). However, our interpretation of the parameter estimates we obtain again depends on this assumption. If it does not hold, then the parameter we call *I*_m_ really represents the ratio *I*_m_/*I*_p_, and the parameters we call *R*_1_ and/or *R*_2_ really represent composite effects of both paternal gene expression and child's genotype. This complication in parameter interpretetion does not arise if one tries to model imprinting within a more classical (and arguably more natural) framework in which we do not assume any main effects of child's or mother's genotype, but simply assume there is an allele in the child, which is expressed when it originates from one parent, but is silenced when it originates from the other parent [Wilkinson et al., 2007]. Such arguably more intuitive models are shown in Examples 14 and 15.

Weinberg [1999b] used an alternative parameterization to model imprinting. Assuming offspring (but no maternal) genotype effects operate, the Weinberg [1999b] parameterization is shown in Example 17, in comparison to the earlier Weinberg et al. [1998] parameterization (Example 16). Comparing the two, it seems that Weinberg [1999b] allows the imprinting effect to operate only when a child is heterozygous, which is biologically rather unintuitive. This model can be better understood if one considers the parameter *I*_m_ not as a parameter that represents any kind of biological mechanism (such as over- or under-expression of a particular allele) but, rather, simply as a statistical device that allows the penetrance for a heterozygous child to vary according to the parental origin of the “2” allele. This idea might be more naturally expressed through the parameterization in Example 18, in which the baseline penetrance α for a child with genotype 11 is multiplied by a factor *R*_m_ if the child inherits an “2” allele from his/her mother, by *R*_p_ if the child inherits an “2” allele from his/her father, and by *R*_2_ if the child is homozygous. The testing of whether there is an allele that operates differentially according to whether it came from the father or mother is then a test of whether *R*_m_ = *R*_p_ in Example 18 or, equivalently, of *I*_m_ = 1 in Example 17.

Although Weinberg et al. [1998] and Weinberg [1999b] appear to be modeling imprinting in rather diferent ways, it can be shown (derivation not given) that the Weinberg [1999b] parameterization corresponds precisely to the earlier Weinberg et al. [1998] parameterization if we write the parameters of the later parameterization (α′, 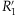, 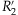, 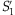, 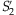, 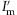, 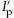) in terms of those of the earlier parameterization as follows: α′ = α, 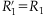, 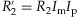, 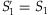, 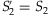, 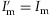, 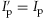. When fitting a null model of no parent-of-origin effects, or an alternative model in which parent-of-origin effects operate, either parameterization will therefore produce the same likelihood and will thus provide equivalent inference regarding the presence or absence of imprinting. *Interpretation* of the resulting parameter estimates, however, will depend on which model one has chosen e.g. is one attempting to model the over-expression of one parental allele, the under-expression of the other parental allele or simply using the imprinting parameter as a statistical device to distinguish the two heterozygote penetrances. Statistically, there is no difference between the different models induced by including either *I*_m_ or *I*_p_, or between the models of Weinberg et al. [1998] and Weinberg [1999b], so any choice between these must be made a priori e.g. based on some prior scientific hypothesis, rather than on statistical grounds.

Example 20 of Table I shows a parameterization used by Li et al. [2009], chosen on the basis of a study by Parimi et al. [2008], who found this parameterization to give generally high power over a range of plausible underlying true models. This parameterization generates a saturated model with seven parameters representing baseline penetrance, additive and dominance effects of child's genotype, additive and dominance effects of mother's genotype, an imprinting parameter that (similar to Weinberg [1999b]) is included only when a child is heterozygous, and an interaction effect that (similar to Sinsheimer et al. [2003]) operates when there is a “mismatch” or “conflict” between maternal and fetal genotype. Since this results in an identifiable saturated model, the parameters of Li et al. [2009] must be expressible as a function of our seven parameters and vice versa. Li et al. [2009] propose using a penalized logistic regression approach to allow parameter estimation even when issues of small sample size induce a collinearity between predictors (even though in theory, given sufficient sample size—leading to observations in all seven cells—all seven parameters should all be identifiable using standard (unpenalized) logistic regression).

The above paragraphs illustrate that, given lack of orthogonality between parameters of a statistical model, when fitting different parameterizations, interpretation of the resulting parameter estimates can be complex. Essentially, the interpretation comes down to how one wishes to define the “baseline” and “effects” of interest. A similar issue has previously been discussed in relation to modeling gene-gene interactions by Cordell [2009]. With respect to actually fitting the models, convergence can readily be achieved either by choosing to include a set of parameters that is known to be identifiable or by using penalization techniques as proposed by Li et al. [2009]. However, successful fitting of the model does not resolve the issue of *interpretation* of the parameter estimates obtained and whether they in fact represent the biological effects that one wishes to encode. This is an issue that is hard (if not impossible) to resolve statistically; rather one needs to start a priori with an underlying biological hypothesis that will dictate the desired form of the model. This might perhaps be informed (or at least better explored) using data from other types of experiment such as gene expression studies. In the Results section, we illustrate some of these difficulties in interpretation via an analysis of a specific example data set, which also serves to demonstrate some unsuspected equivalences between several of the models described in this section.

### RELATIVE RISKS IN CASES VS. CONTROLS, COMPARED TO MOTHERS OF CASES VS. MOTHERS OF CONTROLS

As an initial step toward disentangling these kinds of complex genetic effects in samples of mothers and their offspring, we investigated the genotype relative risks arising from various underlying genetic models when analyzing SNP data in either cases vs. controls or mothers of cases vs. mothers of controls. Table II gives various probabilities that are required for the derivation of the genotype relative risks. In Appendix A, we use Table II to derive formulae for the apparent genotype relative risks when analyzing cases vs. controls, or mothers of cases vs. mothers of controls, under various models. Special cases of these formulae (when only maternal, fetal or imprinting effects operate in isolation) were given by Buyske [2008]. Our results generalize the results of Buyske [2008] to more complex scenarios, and are consistent with his results for the special cases he considered.

**Table II:** Multinomial probabilities for genotype combinations in case/parent trios

Column index									

1	2	3	4	5	6	7	8	9	10

Cell (row) index	Genotypes^a^*g*_m_*g*_f_*g*_c_	Index of parental mating type	*P*(dis|*g*_m_, *g*_f_, *g*_c_)^b^^c^	*P*(*g*_c_|*g*_m_, *g*_f_)^c^	*P*(*g*_m_, *g*_f_)^c^^d^ assuming random mating and HWE	*P*(*g*_m_, *g*_f_)^c^ assuming only exchangeable parental mating types	*P*(*g*_m_, *g*_f_, *g*_c_|dis)^b^^c^^e^ assuming only exchangeable parental mating types	*P*(*g*_m_, *g*_f_, *g*_c_|dis)^b^^c^^e^ with reparameterization Σ = *K*/α µ_1_ = 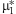 /Σ µ_2_ = 0.5 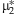 /Σ µ_3_ = 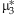 /Σ µ_4_ = 0.25 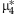 /Σ µ_5_ = 0.5 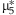 /Σ µ_6_ = 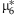 /Σ	*P*(*g*_m_, *g*_f_, *g*_c_|dis)^b^^c^ with reparameterization 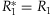 *I*_p_ 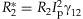 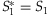 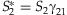 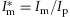 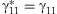 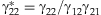
1	22 22 22	1	α*R*_2_*S*_2_*I*_m_*I*_p_γ_22_	1	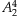	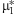	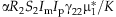	*R*_2_*S*_2_*I*_m_*I*_p_γ_22_µ_1_	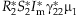
2	22 12 22	2	α*R*_2_*S*_2_*I*_m_*I*_p_γ_22_	0.5	2 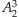 *A*_1_	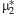	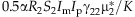	*R*_2_*S*_2_*I*_m_*I*_p_γ_22_µ_2_	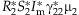
3	22 12 21	2	α*R*_1_*S*_2_*I*_m_γ_21_	0.5	2 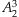 *A*_1_	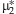	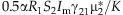	*R*_1_*S*_2_*I*_m_γ_21_µ_2_	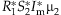
4	12 22 22	2	α*R*_2_*S*_1_*I*_m_*I*_p_γ_12_	0.5	2 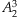 *A*_1_	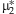	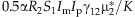	*R*_2_*S*_1_*I*_m_*I*_p_γ_12_µ_2_	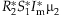
5	12 22 12	2	α*R*_1_*S*_1_*I*_p_γ_11_	0.5	2 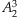 *A*_1_	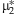	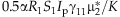	*R*_1_*S*_1_*I*_p_γ_11_µ_2_	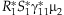
6	22 11 21	3	α*R*_1_*S*_2_*I*_m_γ_21_	1	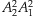	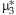	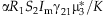	*R*_1_*S*_2_*I*_m_γ_21_µ_3_	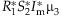
7	11 22 12	3	α*R*_1_*I*_p_	1	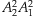	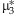	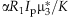	*R*_1_*I*_p_µ_3_	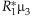
8	12 12 22	4	α*R*_2_*S*_1_*I*_m_*I*_p_γ_12_	0.25	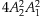	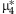	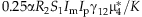	*R*_2_*S*_1_*I*_m_*I*_p_γ_12_µ_4_	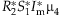
9a	12 12 12	4	α*R*_1_*S*_1_*I*_p_γ_11_	0.25	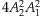	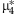	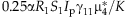	*R*_1_*S*_1_*I*_p_γ_11_µ_4_	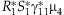
9b	12 12 21	4	α*R*_1_*S*_1_*I*_m_γ_11_	0.25	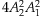	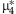	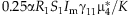	*R*_1_*S*_1_*I*_m_γ_11_µ_4_	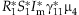
10	12 12 11	4	α*S*_1_	0.25	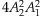	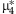	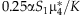	*S*_1_µ_4_	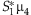
11	12 11 21	5	α*R*_1_*S*_1_*I*_m_γ_11_	0.5	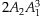	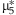	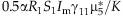	*R*_1_*S*_1_*I*_m_γ_11_µ_5_	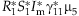
12	12 11 11	5	α*S*_1_	0.5	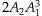	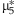	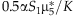	*S*_1_µ_5_	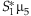
13	11 12 12	5	α*R*_1_*I*_p_	0.5	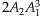	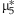	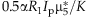	*R*_1_*I*_p_µ_5_	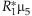
14	11 12 11	5	α	0.5	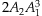	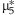	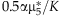	µ_5_	µ_5_
15	11 11 11	6	α	1	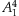	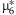	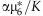	µ_6_	µ_6_

a *g*_m_ and *g*_f_ refer to the unordered alleles in the mother and father respectively. *g*_c_ refers to the ordered alleles (maternal/paternal) in the child.

b dis indicates the event that the child is affected with disease.

c (*g*_m_, *g*_f_) refer to the ordered parental genotypes (maternal, paternal).

d *A*_1_ and *A*_2_ are the allele frequencies of allele 1 and 2, respectively.

e Formulae for *K* and Σ are given in the Appendix.

### A MULTINOMIAL LIKELIHOOD MODEL FOR MOTHER-OFFSPRING DUOS

Next, we extend the approach of Weinberg et al. [1998]; Weinberg [1999b] for case/parent trios to apply instead to case/mother duos. First, we re-derive the Weinberg model for case/parent trios. Column 8 of Table II gives the cell probabilities for the 15 possible outcomes for genotypes of a mother, father and diseased child, *P*(*g*_m_, *g*_f_, *g*_c_|dis), parameterized in terms of the parameters described above, *K* (the population prevalance of disease) and 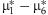 (mating-type stratification parameters as given in column 7 of Table II). The cell probabilities in column 8 are calculated from the probablities in columns 4, 5, and 7 using Bayes' theorem:


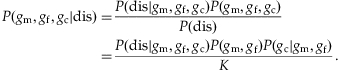


Note that *K* is not a free parameter but rather is a function of the parameters 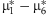 and of 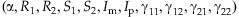 (see Appendix B). We may replace the factor (α/*K*) that appears in each cell of column 8 by a divisor Σ (see Appendix B) that is a function of 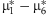 and of 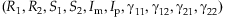.

If we are willing to assume random mating and Hardy-Weinberg equilibrium (HWE), the parameters 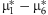 may be written in terms of the allele frequencies *A*_1_ and *A*_2_ (= 1−*A*_1_), as shown in Appendix B and column 6 of Table II. A similar exploitation of an assumption of random mating and HWE was made by Chen et al. [2009], while Childs et al. [2010] assumed random mating but not HWE. Shi et al. [2009] considered several different assumptions, including HWE and random mating. By retaining the parameterization in terms of 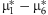, we make the less restrictive assumption of exchangeability of parental mating types [Cordell et al., 2004; Weinberg, 1999b; Weinberg et al., 1998]. The parameters 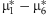 are convenient to work with as they are directly related to the allele frequencies *A*_1_ and *A*_2_ under random mating and HWE; however, they do not correspond precisely to the mating-type stratification parameters used by Weinberg et al. [1998] nor to the slightly different parameterization used by Vermeulen et al. [2009]. By reparameterizing our model as shown in column 9 of Table II, we obtain the same parameterization as in Table V of Weinberg et al. [1998], except that Weinberg et al. [1998] did not consider maternal-fetal interactions and so the parameters γ_*ij*_ do not appear in their formulation.

The model for the cell probabilities as given in column 9 is overparameterized and thus not all of these parameters will be statistically identifiable. By reparameterizing as shown in column 10, we obtain seven identifiable relative risk parameters 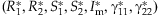 [Cordell et al., 2004]. (Note this is one more parameter of interest than is identifiable using data of the form shown in Table I: in Table I, we can identify up to seven parameters including the baseline disease risk, i.e. up to six relative risk parameters of interest.) This reparameterization can be thought of as being equivalent to a model in which we estimate the original parameters 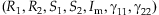 while setting *I*_p_, γ_12_, and γ_21_ equal to 1. (If this assumption is not correct, then the parameters we estimate will in fact correspond to composites of various other parameters, see earlier discussion). If preferred, we could instead set *I*_m_ to 1 and estimate *I*_p_, and/or estimate some alternative set of interaction and main effect parameters. Weinberg et al. [1998] and Weinberg [1999b] model the cell probabilities in column 10 via the use of log-linear models. We propose instead to fit the model by direct maximization of a multinomial likelihood. The equivalence between log-linear (Poisson) and multinomial models [Baker, 1994] implies that these two approaches should provide equivalent inference. Although slightly more computationally intensive, we find the multinomial modeling approach to be more convenient for the following reason: Note that the rows indexed 9a and 9b in Table II correspond to the situation in which the child and both parents are heterozygous, and thus the parent-of-origin of the child's high-risk allele is unknown. We must therefore fit a multinomial likelihood model with 15 possible outcomes (corresponding to the 15 possible observed cells), in which the probability of outcome 9 is assumed equal to the sum of the probabilities in cells 9a and 9b. Log-linear models in which certain cell probabilities correspond to sums of products (rather than purely products) of parameters of interest are difficult to fit in standard statistical software. The log-linear modeling approach to this issue [Weinberg et al., 1998] therefore considers the true outcome (9a or 9b) as missing data, and maximizes the likelihood via use of an EM algorithm. This approach has been implemented in specialist software packages such as LEM [van Den Oord and Vermunt, 2000]. By directly maximizing the multinomial likelihood (e.g. via a direct search algorithm), we avoid the issue of having to fit log-linear models in which certain cell probabilities correspond to sums of the parameters of interest (via an EM algorithm or some other means), an issue that becomes all the more important when we consider collapsing the table down yet further, as described below.

If data are available only on mothers and affected offspring, the 15 observable cells of Table II may be collapsed down to seven observable cells [Weinberg and Shi, 2009], as shown in Table III. To fit models and perform parameter estimation, we propose fitting a multinomial likelihood to the data in these seven cells, leading to a maximum of six estimable parameters (since the total of the cell probabilities must add up to 1). Although ideally one would like to fit the model as parameterized in columns 3 and 4 of Table III, this model is overparameterized and so we instead propose assuming HWE and random mating in order to fit the model as shown in column 5 of Table III. To reduce the number of parameters to estimate, one possibility is to fix the allele frequency *A*_2_ = 1−*A*_1_ at some external value, estimated, for example, from external population-based studies or from published data. (In our simulation study below we assess the sensitivity of our method to misspecification of this value). Since only six parameters are estimable from the data in column 5 of Table II, this model is still over-parameterized and at least one of the parameters (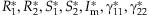) must be set equal to 1. We may in addition choose to set further parameters equal to 1, in order to allow estimation of the allele frequency *A*_2_, rather than fixing it at some external value.

**III:** Multinomial probabilities for genotype combinations in case/mother duos

1	2	3	4	5

Cell (row) index	Genotypes^a^*g*_m_*g*_c_	*P*(*g*_m_, *g*_c_|dis)^b^	*P*(*g*_m_, *g*_c_|dis)^b^ with reparameterization 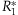 = *R*_1_*I*_p_ 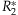 = *R*_2_ 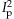 γ_12_ 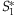 = *S*_1_ 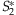 = *S*_2_γ_21_ 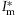 = *I*_m_/*I*_p_ 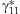 = γ_11_ 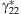 = γ_22_/γ_12_γ_21_	*P*(*g*_m_, *g*_f_, *g*_c_|dis)^b^^c^^d^ assuming random mating and HWE (and with reparameterization from column 4)
1	22 22	*R*_2_*S*_2_*I*_m_*I*_p_γ_22_(µ_1_+µ_2_)	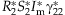 (µ_1_+µ_2_)	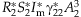 /Σ
2	22 12	*R*_1_*S*_2_*I*_m_γ_21_(µ_2_+µ_3_)	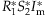 (µ_2_+µ_3_)	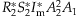 /Σ
3	12 22	*R*_2_*S*_1_*I*_m_*I*_p_γ_12_(µ_2_+µ_4_)	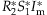 (µ_2_+µ_4_)	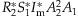 /Σ
4	12 12	*R*_1_*S*_1_γ_11_(*I*_m_(µ_4_+µ_5_)*+I*_p_(µ_2_+µ_4_))	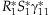 ( 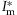 (µ_4_+µ_5_)+µ_2_+µ_4_)	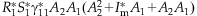 /Σ
5	12 11	*S*_1_(µ_4_+µ_5_)	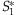 (µ_4_+µ_5_)	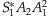 /Σ
6	11 12	*R*_1_*I*_p_(µ_3_+µ_5_)	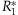 (µ_3_+µ_5_)	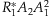 /Σ
7	11 11	µ_5_+µ_6_	µ_5_+µ_6_	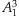 /Σ

a *g*_m_ refers to the unordered alleles in the mother. *g*_c_ refers to the unordered alleles in the child.

b dis indicates the event that the child is affected with disease.

c *A*_1_ and *A*_2_ are the allele frequencies of allele 1 and 2, respectively.

d Formula for Σ is given in the Appendix.

In order to avoid making the assumption of HWE and random mating and/or having to pre-specify a fixed value for *A*_2_, we may assume the additional availability of one or more additional control samples. We consider three possible control samples that might be conveniently utilized: a sample of unrelated controls [Epstein et al., 2005; Nagelkerke et al., 2000], a separate sample consisting of the parents of (additional) unrelated controls [Weinberg and Umbach, 2005], and a further separate sample consisting of (additional) mother-offspring duo controls (i.e. unrelated controls plus their mothers) [Vermeulen et al., 2009]. Note that unrelated controls can provide information on the allele frequency *A*_2_, allowing one to fit the model of column 5 in Table III, while parents of controls and/or mother-offspring duo controls can be used to provide information on the mating-type stratification parameters µ_1_−µ_6_, allowing one to fit the model of column 4 in Table III. The control samples may be incorporated into the multinomial likelihood approach by multiplying the original likelihood by an additional multinomial likelihood for each separate control sample, in which the observed cell counts (genotypes of controls, mating types of parents of controls and/or genotype combinations for mother-offspring duo controls) are written in terms of the allele frequency *A*_2_ (if assuming HWE and random mating) or else in terms of the parameters 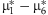 (or, equivalently, µ_1_−µ_6_). Supplementary Tables I–III show the relevant cell probabilities corresponding to these multinomial likelihoods, assuming the control sample comprises a random sample from the population (i.e. of unknown disease status). Equivalent results would be obtained from genuinely unaffected controls on the condition that the disease is rare. (If the disease is common, unaffected controls would not be suitable for this analysis as they lead to tables with a slightly different structure [Weinberg and Shi, 2009].)

Weinberg and Umbach [2005] investigated the incorporation of parents of controls into their log-linear modeling approach for case/parent trios (noting that no additional information for estimation of µ_1_−µ_6_ is provided by genotyping the controls themselves, once their parents have been genotyped). They showed that this hybrid design can improve the efficiency for estimation of the main effects of maternal and child genotype, compared to using a case/parent trio or case/mother vs. control/mother approach. Vermeulen et al. [2009] considered incorporation of control/mother duos into the log-linear modeling approach when performing a joint (4 df) test of offspring and maternal effects; they found this design improved power for testing the null hypothesis that all effects are equal to 0. However, Vermeulen et al. [2009] did not investigate parameter estimation or the issue of testing of more complex hypotheses (such as the hypothesis that maternal effects exist but offspring effects do not) nor did they allow for the existence of parent-of-origin effects or mother-child interactions. Our investigation therefore complements the investigations performed in these earlier studies.

### SIMULATION STUDY

We conducted computer simulations to assess the performance (parameter estimation, type 1 error and power) of our multinomial likelihood modeling approach for case/mother duos, with or without the inclusion of a separate control sample as described above. We considered ten different scenarios, labeled A–J. The true parameter values used in each scenario are shown in Table IV. Data (500 case/mother duos, together with 500 units of any other required data structure e.g. control/mother duos) were simulated under the relevant parameter settings and analyzed using our approach (maximization of the product of the relevant multinomial likelihoods), incorporating different (full and reduced) sets of parameters in the model as required. We compared our results to standard logistic regression analysis of case/mother duos vs. control/mother duos with the same set of parameters incorporated as predictor variables. Table V shows the different methods (i.e. parameter restrictions and availability of control samples) we considered. In addition, we investigated the performance of our multinomial likelihood approach when applied to 500 case/parent trios (i.e. assuming fathers were available), either using the full parameterization as shown in the column 10 of Table II (thus equivalent to Weinberg's log-linear modeling approach), or by assuming HWE and random mating to rewrite the parameters 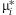, and thus the µ_*i*_, in terms of the allele frequency *A*_2_. The advantage of making these assumptions compared to the original Weinberg approach (in which mating-type stratification parameters µ_1_−µ_6_ are freely estimated) is that it reduces the number of parameters to estimate; however, the resulting method may not retain its robustness to population stratification.

**Table IV:** Parameter values assumed under simulation scenarios A–J

	Parameter value									
	
Scenario	*A*_2_	α	*R*_1_	*R*_2_	*S*_1_	*S*_2_	*I*_m_	*I*_p_	γ_11_	γ_22_
A	0.3	0.1	1.5	2.25	1	1	1	1	1	1
B	0.3	0.1	1	1	1.5	2.25	1	1	1	1
C	0.3	0.1	1.5	2.25	1.5	2.25	1	1	1	1
D	0.3	0.1	1.5	2.25	1	1	1.8	1	1	1
E	0.3	0.1	1.5	2.25	1	1	1	1.8	1	1
F	0.3	0.1	1.5	2.25	1.5	2.25	1.8	1	1	1
G	0.3	0.1	1.5	2.25	1.5	2.25	1	1.8	1	1
H	0.3	0.1	1.5	2.25	1.5	2.25	1	1	0.5	0.5
I	0.3	0.1	1.5	2.25	1.5	2.25	1.8	1	0.5	1
J	0.3	0.1	1.5	2.25	1.5	2.25	1	1.8	0.5	1

**Table V:** Methods evaluated in simulation study

Method	Description	Additional control samples used	Assumptions	Parameters estimated in addition to disease risk parameters
0	Logistic regression of case/mother duos versus control/mother duos	Control/mother duos used (by definition)	None	None
1	Multinomial model	None	HWE+RM+Fixed allele frequency *A*_2_	None
2	Multinomial model	None	HWE+RM	*A*_2_
3	Multinomial model	Controls	HWE+RM	*A*_2_
4	Multinomial model	Parents of controls	HWE+RM	*A*_2_
5	Multinomial model	Parents of controls	Parental allelic exchangeability	µ_1_*−*µ_6_ with µ_3_ = µ_4_
6	Multinomial model	Parents of controls	Mating symmetry	µ_1_−µ_6_
7	Multinomial model	Control/mother duos	HWE+RM	*A*_2_
8	Multinomial model	Control/mother duos	Parental allelic exchangeability	µ_1_−µ_6_ with µ_3_−µ_4_
9	Multinomial model	Control/mother duos	Mating symmetry	µ_1_−µ_6_

HWE, Hardy-Weinberg equilibrium; RM, random mating.

## RESULTS

### RELATIVE RISKS IN CASES VS. CONTROLS COMPARED TO MOTHERS OF CASES VS. MOTHERS OF CONTROLS

Table VI shows the general formulae for genotype relative risks in cases vs. controls compared to mothers of cases vs. mothers of controls, as calculated in Appendix A. Specific examples of the resulting genotype relative risks under different scenarios are shown in Supplementary Table IV. Although only a limited number of scenarios are examined in Supplementary Table IV, nevertheless we may make the following observations:

**Table VI:** Formulae for GRRs in cases versus controls and in mothers of cases versus mothers of controls (GRRs calculated assuming Hardy-Weinberg Equilibrium and random mating)

	GRRs	
	
Sample	RR_12_	RR_22_
>Cases vs. controls	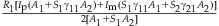	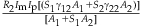
Mothers of cases vs. mothers of controls	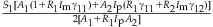	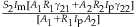

GRRs, genotype relative risks.

1. If the true model is due to effects of child's own genotype, then the relative risks when analyzing mothers of cases vs. of mothers of controls are attenuated compared to the true risks, both in absolute value and in their pattern of inheritance, in the sense that a dominant model in the child appears closer to a recessive model in the mother, and a recessive model in the child appears closer to a dominant model in the mother. This phenomenon is a classic case of confounding, whereby child genotype effects are misattributed to the mother, when the child's own genotype is not included in the model.
2. If the true model is due to effects of the mother's genotype, then the relative risks in cases vs. controls are attenuated, both in absolute value and in their pattern of inheritance, in the sense that a dominant model in the mother appears closer to recessive in the child, and a recessive model in the mother appears closer to dominant in the child. The power implications of the attenuation in relative risks are illustrated in the final two columns of Supplementary Table IV, which show the resulting power (calculated using the Genetic Power Calculator [Purcell et al., 2003]) obtained from 500 cases vs. controls compared to when using 500 mothers of cases vs. mothers of controls. Analysis under the incorrect model (e.g. using cases and controls when the effect is really due to the mother's genotype) can lead to a considerable decrease in power e.g. (see row 7) from 97% under the correct model to only 39% under the incorrect model.
3. The same pattern of relative risks in a child could arise from a variety of different mechanisms. For example, relative risks *RR*_12_ = 1.5 and *RR*_22_ = 2 in the child could arise from a mother's genotype effect (*S*_1_ = 2, *S*_2_ = 4), a maternal imprinting effect (*I*_m_ = 2), a paternal imprinting effect (*I*_p_ = 2), or simply from the child's own genotype (*R*_1_ = 1.5, *R*_2_ = 2). These different scenarios could be distinguished by additionally looking at the genotype relative risks in mothers of cases vs. of mothers of controls.
4. A maternal imprinting effect (*I*_m_>1) will be visible (and will lead to identical genotype relative risks) when analyzing either cases vs. controls or mothers of cases vs. of mothers of controls. A paternal imprinting effect, on the other hand, will be visible when analyzing cases vs. controls but will lead to no increase in risk when considering mothers of cases vs. of mothers of controls.

### MULTINOMIAL MODELING OF CASE/MOTHER DUOS

We used computer simulations to investigate the performance of our multinomial modeling approach. Our approach has been implemented in a freely available Fortran program EMIM (**E**stimation of **M**aternal, **I**mprinting and interaction effects using **M**ultinomial modelling). Data were simulated under scenarios A–J and were analyzed using either logistic regression (Method 0) or EMIM (Methods 1−9) under various different assumptions concerning the parameters estimated and the availability of additional control samples (see Table V). In addition to investigating the performance of the methods under assumptions of either HWE and random mating or mating symmetry (which allows estimation of the six mating-type stratification parameters µ_1_−µ_6_ [Weinberg, 1999b; Weinberg et al., 1998], we also investigated the use of an alternative parental allelic exchangeability assumption [Shi et al., 2008], which, in context of the parameterization here, corresponds to assuming that µ_4_−µ_3_.

**Fig. 1:**
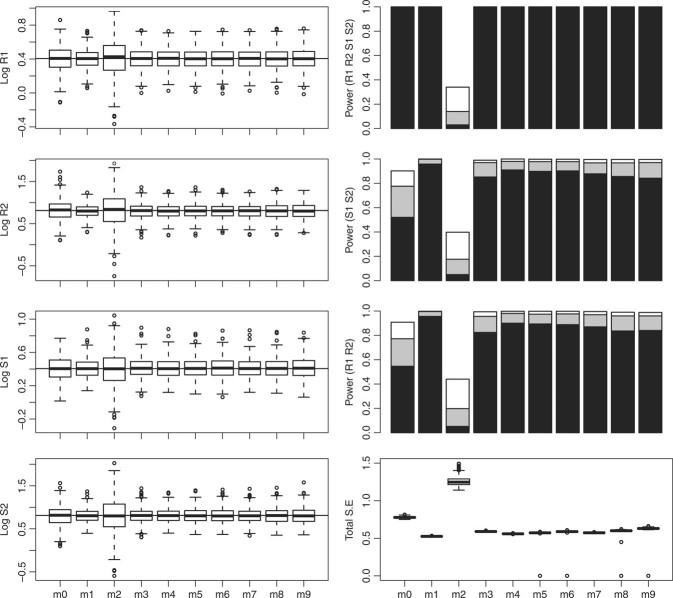
Results from simulation scenario C. The different methods are denoted m_0_–m_9_. Five hundred case/mother duos were simulated, together with various control samples (500 unrelated controls, 500 units each consisting of the two parents (mother and father) of a control, or 500 control/mother duos) for use in Methods 3–9. The left panels show boxplots of the parameter estimates (logs of the given relative risk parameter) over 500 simulation replicates. A horizontal line is drawn at the true value of the log of the given parameter. The top three right panels show the power of likelihood ratio tests of various hypotheses. Power to achieve significance levels (*P* values) of 0.05, 0.01, and 0.001 are shown in white, gray, and black, respectively. The top panel shows the power for testing the full model (*R*_1_, *R*_2_, *S*_1_, and *S*_2_) against a null model where all parameters equal 1. The second panel shows the power for testing the full model (*R*_1_, *R*_2_, *S*_1_, and *S*_2_) against a null model that includes *R*_1_ and *R*_2_ only (i.e. the power for detecting the maternal genotype effects *S*_1_ and *S*_2_ while allowing for child genotype effects). The third panel shows the power for testing the full model (*R*_1_, *R*_2_, *S*_1_, and *S*_2_) against a null model that includes *S*_1_ and *S*_2_ only (i.e. the power for detecting the child genotype effects *R*_1_ and *R*_2_ while allowing for maternal genotype effects). The bottom right panel shows a boxplot of the total estimated standard error (SE) (i.e. the sum of the estimated standard errors of all four estimated parameters) over the 500 simulation replicates.

**Fig. 2:**
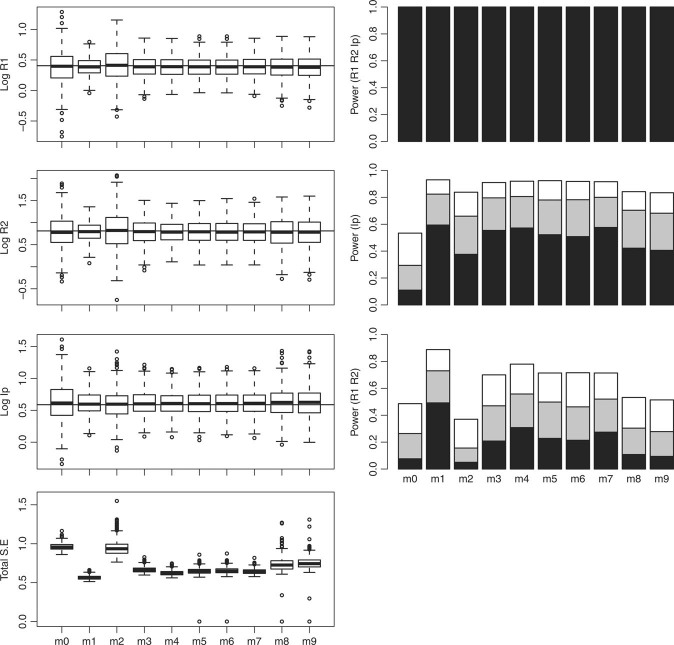
Results from simulation scenario E. See figure legend to Figure 1 for detailed description of plots. Here the top three left hand panels show boxplots of the relevant parameter estimates (logs of the given relative risk parameter) with a line indicating the true value, and the top three right hand panels show the power of likelihood ratio tests of various hypotheses (specifically of whether the given parameter(s) are zero, when allowing for the effects of the other parameters). The lowest left panel shows a boxplot of the total estimated standard error (SE) over the 500 simulation replicates.

Figures 1–3 show the results (boxplots of the individual parameter estimates, the sum of the estimated standard errors for all parameters and the power for testing various hypotheses) evaluated over 500 simulation replicates for scenarios C, E, and F. In scenario C, the risk of disease depends on the maternal and child genotypes but there are no imprinting or interaction effects. In scenario E, the risk of disease depends on the child's genotype together with an imprinting effect. In scenario F, the risk of disease depends on maternal and child genotypes together with an imprinting effect. Results for other scenarios are shown in Supplementary Figures 1–7(with interaction effects included in scenarios H, I, and J).

**Fig. 4:**
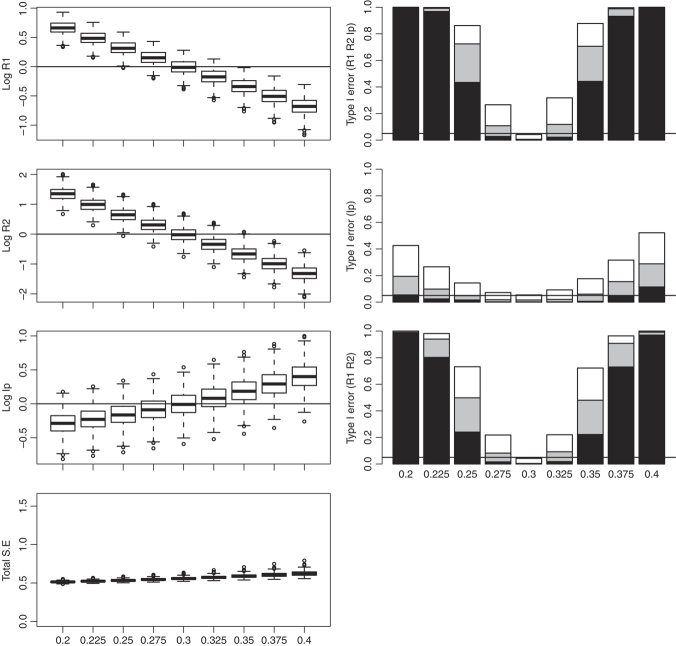
Sensitivity to misspecification of minor allele frequency *A*_2_. See figure legend to Figure 1 for detailed description of plots. Results are shown for method 1 with minor allele frequency *A*_2_ assumed to be either 0.2, 0.225, 0.25. 0.3, 0.325, 0.35, 0.375 or 0.4. The true value of *A*_2_ used in the simulation was 0.3. Three parameters (*R*_1_, *R*_2_, and *I*_m_) were fitted according to scenario E (similar results were found for other scenarios). Data were simulated under the global null, i.e. the true value of each of these parameters was 1. The top left panels show boxplots of the parameter estimates (logs of the given relative risk parameter) with a line indicating the true value, the lowest left panel shows a boxplot of the total estimated standard error (SE) and the three right panels show the type 1 error for likelihood ratio tests of whether the given parameter or parameters are equal to 1. Type 1 errors for nominal significance levels (*P* values) of 0.05, 0.01, and 0.001 are shown in white, gray, and black, respectively.

**Fig. 5:**
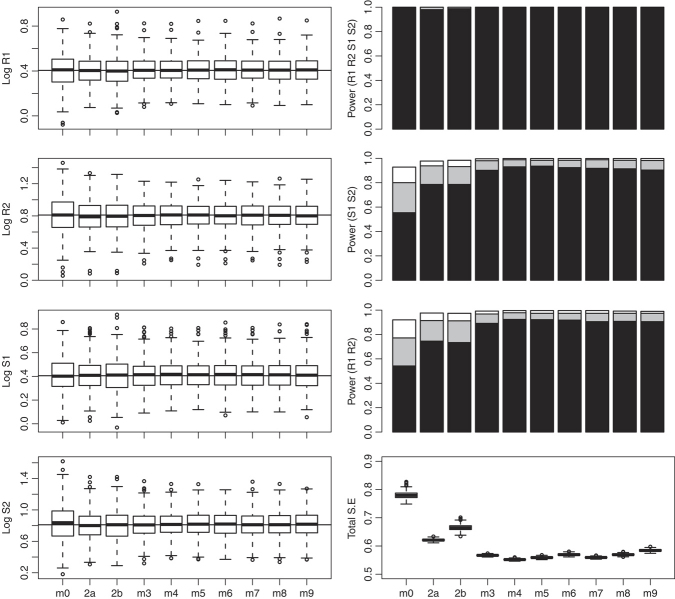
Results from simulation scenario C, case/parent trios. See figure legend to Figure 1 for detailed description of plots. Method 1 was not considered, but two different versions of Method 2 (2a and 2b) were considered, as described in the text.

**Fig. 6:**
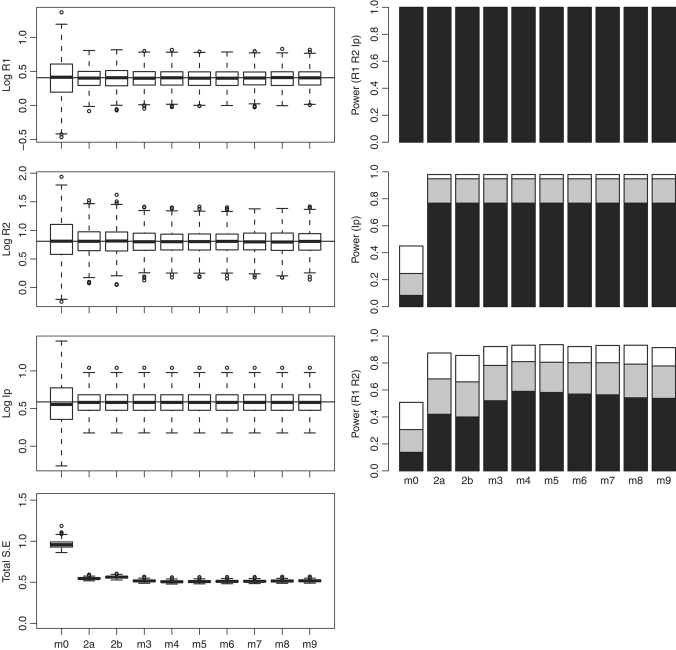
Results from simulation scenario E, case/parent trios. See figure legend to Figures 1 and 2 for detailed description of plots. Method 1 was not considered, but two different versions of Method 2 (2a and 2b) were considered, as described in the text.

**Fig. 7:**
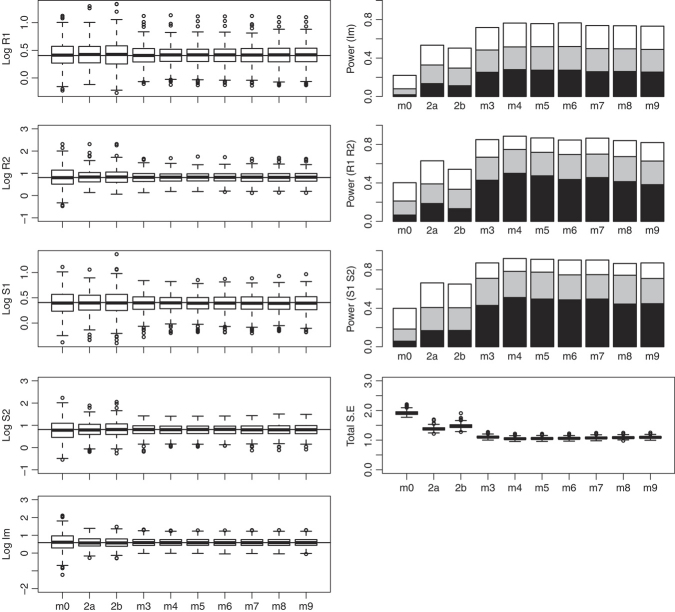
Results from simulation scenario F, case/parent trios. See figure legend to Figures 1 and 3 for detailed description of plots. Method 1 was not considered, but two different versions of Method 2 (2a and 2b) were considered, as described in the text.

In all scenarios, we find unbiased estimation of the parameters, although the variability in the parameter estimates (over the 500 replicates), along with the total estimated standard error, increases as the models become more complex (as larger numbers of parameters are estimated). As illustrated by Figures 1–3 (and Supplementary Figures 1–7), Method 0 (logistic regression) and Method 2 (in which the allele frequency *A*_2_ is estimated from the case/mother duo sample alone) perform the most poorly of all methods considered, with largest variability in the parameter estimates, largest total estimated standard error, and lowest power. Method 1, in which the allele frequency *A*_2_ is fixed to its true value, performs best, with the lowest variability in the parameter estimates, smallest total estimated standard error, and the highest power. Method 3, in which a separate sample of controls is used to help estimate *A*_2_, performs slightly worser than Method 1. Performance can be improved by using instead parents of controls (giving a sample size twice as large as with controls) to help estimate *A*_2_ under the assumption of HWE and random mating (Method 4). Methods 4–6 all use a sample of parents of controls but under progressively less restrictive assumptions (HWE and random mating, parental allelic exchangeability and mating symmetry, respectively), resulting in a progressively worse performance (lower power, higher variability, and total standard error) as less assumptions are made and more parameters are estimated. Note, however, that our data were simulated to accord with the assumption of HWE and random mating, which provides a “best case” scenario for Method 4. The advantage of Methods 5 and 6 is that they should be robust to violations of HWE and random mating, which will not be true of Method 4.

Methods 7–9 all use a sample of control/mother duos to help estimate either *A*_2_ or µ_1_−µ_6_, again under progressively less restrictive assumptions. Again this results in a progressively worse (but presumably more robust) performance as more parameters are estimated. For a given assumption (HWE and random mating, parental allelic symmetry or mating symmetry), parents of controls appear to provide better performance than an equal number of control/mother duos, as found by Vermeulen et al. [2009].

Although Method 1 performs best, it makes the very restrictive assumption that we can fix the allele frequency *A*_2_ at its true value, without allowing for any variability associated with an estimate of *A*_2_. We investigated the effect of misspecification of *A*_2_. We simulated data under the null hypothesis of no genetic effects 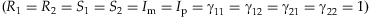 and analyzed the data using Method 1 with five parameters estimated 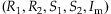 as in scenario F. The true minor allele frequency *A*_2_ was set at 0.3, but in the analysis we assumed values of *A*_2_ between 0.2 and 0.4. Figure 4 shows the resulting parameter estimation and type 1 error. Provided *A*_2_ was correctly specified at 0.3, parameter estimation was unbiased and type 1 error remained at nominal levels. However, misspecification of *A*_2_ resulted in biased parameter estimation (either upwards or downwards) and high type 1 error. Therefore, unless the allele frequency is accurately known, it would seem safer to incorporate an additional control sample into the analysis to aid in its estimation (as in Methods 3–9). We checked the type 1 error and parameter estimation of all other methods under the null for scenarios C and F using a larger number of simulation replicates (Supplementary Figures 8 and 9) and found, as expected from standard statistical theory of likelihood ratio tests, that nominal significance levels were maintained. Similar results were found under other scenarios (data not shown).

**Fig. 8:**
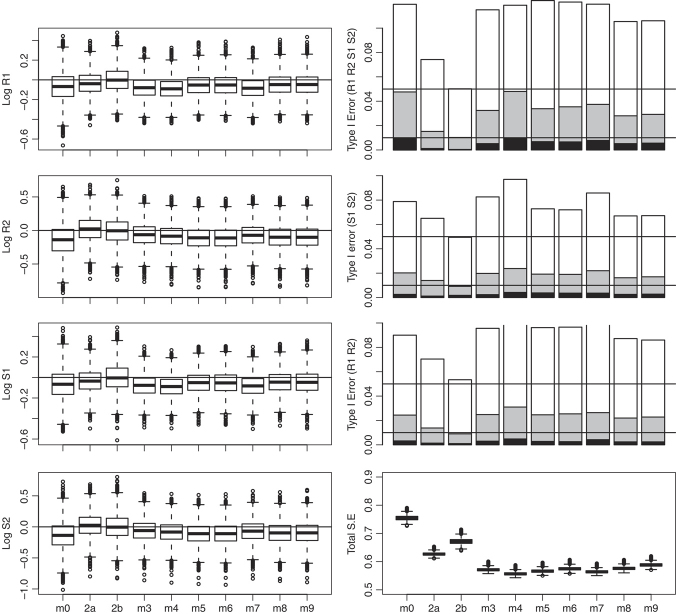
Results from simulation scenario C, case/parent trios with population stratification. Results are from 5,000 simulation replicates. See figure legend to Figures 1,5 and Supplementary Figure 8 for detailed description of plots. Data were simulated under the null hypothesis of no genetic effects, but in the presence of population stratification (two different sub-populations with differing disease rates and marker allele frequencies).

**Fig. 9:**
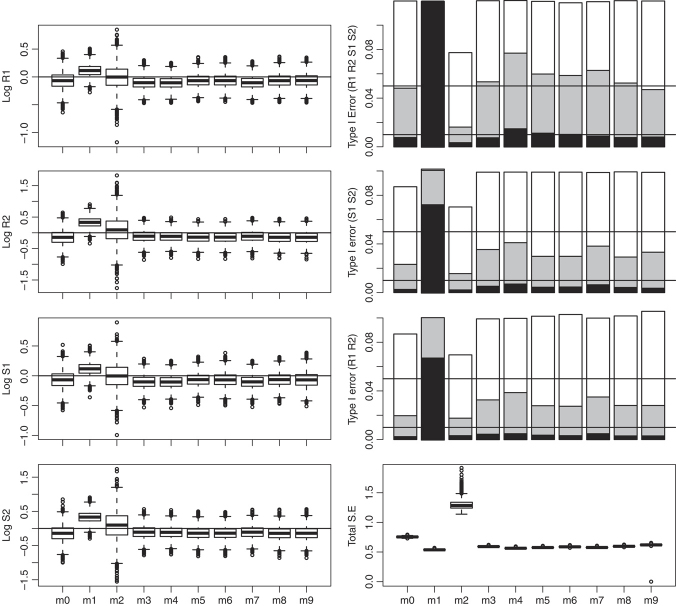
Results from simulation scenario C, case/mother duos with population stratification. Results are from 5,000 simulation replicates. See figure legend to Figure 1 and Supplementary Figure 8 for detailed description of plots. Data were simulated under the null hypothesis of no genetic effects, but in the presence of population stratification (two different sub-populations with differing disease rates and marker allele frequencies).

We also investigated the effect of sample size on the different methods, using simulation scenario G, which aims to estimate five parameters (*R*_1_, *R*_2_, *S*_1_, *S*_2_, and *I*_p_). Our previous simulations assumed the availability of 500 case/mother duos, together with an equal number of unrelated controls, parents (i.e. 500 units each consisting of the mother and father) of controls or control/mother duos (for use in Methods 3–9). Supplementary Figure 10 shows the total estimated standard error and power for the different methods as the sample size (number of observations of each type) varies between 100 and 500. With respect to power, for the parameter values assumed here, a sample size of around 300 case/mother duos seemed to be required to give reasonable power for detection of effects. At lower sample sizes, several of the methods have occasional problems with estimation of certain parameters (resulting in a total standard error output value of 0, indicating that the parameter and/or its variance was not able to be estimated). These problems generally corresponded to simulation replicates in which one or more of the cells in the relevant tables of counts (Table II, Supplementary Tables 1–3) had zero entries. Not surprisingly, given insufficient data, it is not always possible to fit complex models with many parameters. To fit the models in this situation, one would need to reduce the number of parameters that one tries to estimate, either manually, or perhaps by use of some kind of penalization approach [Li et al., 2009].

**Fig. 10:**
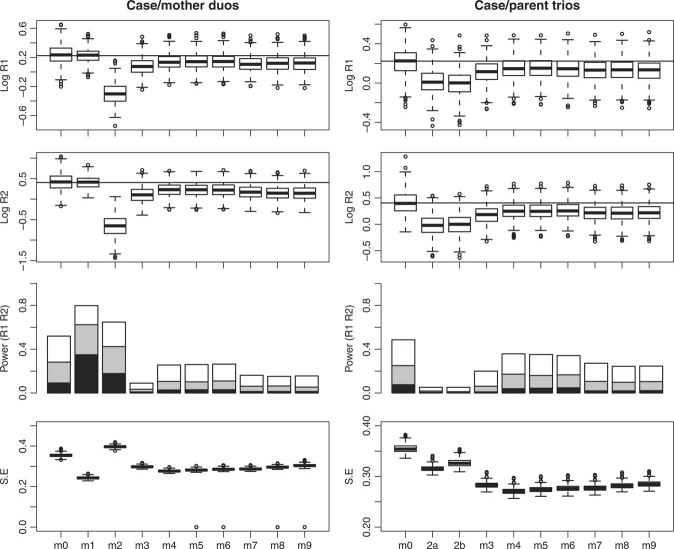
Results from data generated under simulation scenario B but analyzed assuming scenario A. See figure legend to Figure 1 for detailed description of plots. A horizontal line is drawn at the expected value of the log of the given parameter using logistic regression, as calculated in Tables VI and VII.

**Table VII:** Parameter estimates and log likelihoods from fitting various models to a single example data set

		Maximum likelihood estimates of parameters representing effects due to																				
					
		Child's genotype				Mother's genotype				Imprinting				Maternal/fetal interactions								
								
Row index	Model fitted	*R*_1_	*R*_2_	ρ_1_	ρ_1_	*S*_1_	*S*_2_	η_1_	η_2_	*I*_m_	*I*_p_	Later *I*_m_	Later *I*_p_ = *j*_m_	γ_11_	γ_22_	γ_01_ = µ_0_	γ_21_ = µ_2_	µ	*j*_c_	Minus null loglik	Minus max loglik	Twice diff in logliks
1	EMIM imprinting (original *I*_m_)	3.86	24.43	–	–	1.16	1.50	–	–	0.35	–	–	–	–	–	–	–	–	–	4424.31	4186.53	475.56
2	EMIM imprinting (original *I*_p_)	1.35	3.00	–	–	1.16	1.50	–	–	–	2.85	–	–	–	–	–	–	–	–	4424.31	4186.53	475.56
3	EMIM imprinting (later Weinberg [1999b] *I*_m_)	3.86	8.56	–	–	1.16	1.51	–	–	–	–	0.35	–	–	–	–	–	–	–	4424.31	4186.53	475.56
4	EMIM imprinting (later Weinberg [1999b] *I*_p_)	1.35	8.56	–	–	1.16	1.51	–	–	–	–	–	2.86	–	–	–	–	–	–	4424.31	4186.53	475.56
5	Sinsheimer et al. [2003] 1A	–	–	1.24	5.44	–	–	2.00	2.30	–	–	–	–	–	–	4.89	–	–	–	4424.31	4176.68	495.26
6	EMIM 5df (single interaction γ_11_)	5.67	6.81	–	–	1.66	0.82	–	–	–	–	–	–	0.20	–	–	–	–	–	4424.31	4166.90	514.81
7	Palmer et al. [2006]	–	–	1.14	6.81	–	–	1.66	0.82	–	–	–	–	–	–	–	–	0.20	–	4424.31	4166.90	514.81
8	EMIM 5df (single interaction γ_22_)	3.08	14.07	–	–	0.55	0.82	–	–	–	–	–	–	–	0.42	–	–	–	–	4424.31	4197.28	454.07
9	Sinsheimer et al. [2003] 1B	–	–	1.03	5.91	–	–	2.02	1.27	–	–	–	–	–	–	6.20	3.48	–	–	4424.31	4165.14	518.35
10	EMIM (γ_11_ and γ_22_)	6.41	5.89	–	–	2.03	0.71	–	–	–	–	–	–	0.16	1.79	–	–	–	–	4424.31	4165.14	518.35
11	Parami et al. [2008] and Li et al. [2009]	1.50	5.90	–	–	1.11	1.55	–	–	–	–	–	2.16	–	–	–	–	–	1.99	4424.31	4161.08	526.45
12	Palmer et al. [2006] with *I*_p_ added	–	–	0.75	5.89	–	–	2.21	1.55	–	2.15	–	–	–	–	–	–	0.25	–	4424.31	4161.08	526.45
13	EMIM 6 df (*I*_p_ and γ_11_)	3.00	2.74	–	–	2.21	1.54	–	–	–	2.15	–	–	0.25	–	–	–	–	–	4424.31	4161.08	526.45
14	EMIM 6 df (*I*_p_ and γ_22_)	0.77	1.80	–	–	1.25	4.82	–	–	–	5.28	–	–	–	0.17	–	–	–	–	4424.31	4168.48	511.66
15	Sinsheimer [2003] 1A with *I*_*p*_ added	–	–	1.15	5.55	–	–	2.00	2.42	–	1.34	–	–	–	–	3.84	–	–	–	4424.31	4176.24	496.15
16	EMIM saturated (*I*_p_, γ_11_ and γ_22_)	2.13	2.27	–	–	2.04	2.22	–	–	2.82	–	–	–	0.33	0.59	–	–	–	–	4424.31	4160.38	527.86
17	Sinsheimer [2003] 1B with *I*_p_added	–	–	0.69	6.41	–	–	2.04	1.31	–	2.86	–	–	–	–	3.04	5.25	–	–	4424.31	4160.38	527.86

EMIM, Estimation of Maternal, Imprinting and interaction effects using Multinomial modeling.

### MULTINOMIAL MODELING OF CASE/PARENT TRIOS

We repeated our simulations assuming the availability of a sample of case/parent trios rather than case/mother duos. We found similar sensitivity to the choice of fixed allele frequency in Method 1 as we had seen in Figure 4 with case/mother duos (data not shown). With case/parent trios, there is no need to fix the value of the allele frequency *A*_2_ even for complex scenarios (such as scenarios F–J) when no additional control samples are available, as all seven parameters of interest are identifiable from the counts in the column 10 of Table II. For case/parent trios, we therefore did not consider Method 1 any further, but instead we considered two alternative versions of Method 2: in Method 2a, we assumed HWE and random mating in order to estimate the parameter *A*_2_, while in Method 2b, we did not assume HWE and random mating but instead estimated the six mating-type stratification parameters µ_1_−µ_6_. All other methods remained as shown in Table V.

Results from scenarios C, E, and F (using a sample size of 500 trios and an equal number of control observations as required) are shown in Figures 5–7. Results from the other scenarios followed a similar pattern (data not shown). In general, the pattern of results in terms of comparison of the different methods was similar to what had been seen with case/mother duos. The performances of Method 0 (logistic regression) and Methods 2a and 2b (which use only the case/parent trios) are considerably worser than those of Methods 3–9. Method 2a, which assumes HWE and random mating to help estimate *A*_2_, performs better than Method 2b (which makes less assumptions and estimates the six mating-type stratification parameters µ_1_−µ_6_). As with case/mother duos, it seems that the use of additional control samples (in the absence of knowledge of the allele frequency *A*_2_) can considerably improve the estimation of the genetic parameters of interest.

Supplementary Figure 11 shows the results from Scenario G as the sample size (number of observations of each type) varies between 100 and 500. Although the absolute power will clearly depend on sample size, allele frequency, and genetic effect sizes, comparison of Supplementary Figure 11 to Supplementary Figure 10 (as well as comparison of Figs. 5–7 with Figs. 1–3) indicates that case/parent trios provide substantially higher power and better parameter estimation than is provided by case/mother duos.

### EFFECT OF POPULATION STRATIFICATION

We also investigated the performance of the different methods applied to either case/parent trios or case/mother duos in the presence of population stratification (Figs. 8 and 9). Data were simulated assuming families came from one of two (unknown) sub-populations with differing baseline risks of disease (0.1 and 0.05) and differing allele frequencies (0.3 and 0.15) respectively. For case/parent trios, only method 2b maintained nominal type 1 error in the presence of population stratification (Fig. 8). For case/mother duos, no method maintained nominal type 1 error (Fig. 9). Since population stratification induces a lack of HWE, we expect all methods that assume HWE and random mating to be compromised. In addition, it seems that the use of the various control samples to help estimate µ_1_−µ_6_ fails, possibly on account of the different mating-type frequencies (between parents of cases and parents of controls) induced by population stratification.

### USE OF COMMON CONTROLS

Our simulations thus far assumed the same number of units of each type (case/mother duos, case/parent trios, control/mother duos etc.) It is becoming increasingly common to make use of data from a large external control sample such as that generated by WTCCC [2007]. (As illustrated by our simulations above, this approach would not be valid if there was population stratification or if the common controls came from a different population compared to the cases.) Assuming all individuals are generated from the same population, Supplementary Figure 12 shows the results of applying our methods (together with logistic regression) to a sample consisting of either 500 case/mother duos or 500 case/parent trios and 3,000 population-based controls. Only Scenarios A and B were considered, since these are the only scenarios that can be modeled using logistic regression when no mothers of controls are available. Even with this larger control sample, our multinomial modeling approach (assuming HWE and random mating) shows higher power and precision of parameter estimation than does logistic regression. Some slight reduction in power is seen for our method in Scenario B when analyzing 500 case/parent trios under the less restrictive assumptions of parental allelic exchangeability or mating symmetry (as opposed to HWE and random mating).

### EFFECT OF MODEL MISSPECIFICATION

Our simulations thus far have assumed that we analyze our data using the “correct” parameterization (in other words, fitting the same set of parameters that were actually used to generate the data). It is of interest to examine the performance of the methods when we generate data under one model but analyze it under another model. For logistic regression, if only mothers or childs effects are fitted, the resulting parameter estimates should take the form calculated in Tables I and II. (Note that the last four rows of Table II give the expected parameter estimates as estimated from logistic regression analysis of either cases vs. controls, or mothers of cases vs. mothers of controls, under our simulation Scenarios A, B, D, and E, respectively.)

Supplementary Figures 13–18 show the results for case/mother duos when a five-parameter model containing child and maternal genotype effects and an imprinting effect (Scenario F) is fitted, even though the true model used for generating the data is smaller (i.e. consists of only a subset of the parameters). The parameter estimates are all found to be unbiased, including those parameters that were not part of the generating model (whose log relative risks estimates are therefore all centred round zero), and correct type 1 error is maintained for these parameters. Method 2 cannot be used to fit a five-parameter model when case/mother duos (as opposed to case/parent trios) are the unit of analysis; however, we can illustrate the same point for Method 2 (as well as all other methods) by fitting a three- or four-parameter model when the generating model is actually smaller (i.e. consists of only a subset of the parameters), see Supplementary Figures 19–21. Therefore, provided the true model is nested within the analysis model, all methods provide unbiased parameter estimation and adequate control of type 1 error, as expected from standard statistical theory. Similar results were found for case/parent trios (data not shown).

A more interesting question perhaps is what happens when the true model is not nested within the analysis model. Supplementary Figures 22 and 23 show the results of analyzing 500 case/parent trios when data are generated under Scenarios D and E but analyzed assuming Scenario A or Scenario B. Interestingly, when analyzed under Scenario A, all methods give the same “apparent” parameter estimates as expected from Table II, but when analyzed under Scenario B, only logistic regression gives the expected parameter estimates from Table II: all other methods give estimates with differing degrees of bias (and therefore either a decrease or increase in power, depending on the extent of the bias). To investigate the cause of this phenomenon in a simpler scenario, we examined the performance of the methods when applied to either case/mother duos or case/parent trios, when data were generated under Scenario B but analyzed under Scenario A (see Fig. 10). Again, only logistic regression (Method 0) gives the expected parameter estimates from Table II. Methods 3–9 give slightly attenuated relative risk estimates and Methods 2 (for duos) or 2a and 2b (for trios) give log relative risk estimates that are essentially centered round 0. Similar results were found when generating data under Scenario A but analyzing under Scenario B (data not shown).

The reason for this phenomenon can be discovered from a closer inspection of the 15 cell probabilities in Table II. For Method 2b, if data are really generated under Scenario B (in which effects *S*_1_ and *S*_2_ operate), but analyzed under Scenario A (in which effects *R*_1_ and *R*_2_ operate), the only data contributing to the estimation of *R*_1_ and *R*_2_ comes from comparing cell counts within each of the six parental mating types. Mating types 1 and 6 do not contribute to this comparison. From mating type 2 (comparison of cells 2+4 to 3+5), we would estimate that *R*_2_/*R*_1_ = (*S*_2_+*S*_1_)/(*S*_2_+*S*_1_) = 1. From mating type 4 (comparison of cells 8 to 10 and 9a+9b to 10), we would estimate that *R*_2_ = 1 and *R*_1_ = 1. From mating type 5 (comparison of cells 11+13 to 12+14), we would estimate *R*_1_ = (*S*_1_+1)/(*S*_1_+1) = 1. Thus, given the chosen parameterization, estimates of *R*_1_ = 1 and *R*_2_ = 1 (log relative risk estimates of 0) will indeed provide the best fit to the data. If, however, there is additional data helping us to estimate the relative magnitude of the mating-type stratification parameters (Methods 3–9), we can borrow information from contrasts *across* the mating types. In that case, the estimates of *R*_1_ and *R*_2_ become essentially weighted averages of the true parameters (*S*_1_ and *S*_2_) and 1, as the model tries to come up with a set of parameter estimates that best resolve these various (misspecified) contrasts.

This result might seem at first glance slightly alarming, as it would suggest that we may be in danger of having no power to detect any effects under Methods 2/2a/2b, if we choose an incorrect parameterization. However, in practice, if there is reason to suspect complex effects of the kind investigated here, a sensible strategy would be to fit a series of different (possibly nested) models, as proposed by Cordell et al. [2004]. For example, one could use a forward or backward stepwise strategy, or perform model comparison via the Akaike Information Criterion (AIC), in order to identify the single best-fitting model. Given that our previous simulations showed there should be high power to estimate effects when they are correctly modeled, there should be little danger of missing such effects provided such a strategy is used.

Supplementary Figures 24 and 25 show the results of analyzing 500 case/parent trios when data are generated under Scenario G but analyzed assuming Scenario H, or vice versa. We find (Supplementary Figure 24) that a true imprinting effect can masquerade as an apparent interaction effect, as well as altering the estimates of *R*_1_, *R*_2_, and *S*_2_. Similar results (“apparent” interactions induced by an imprinting effect) were found when modeling the interaction term(s) via the MFG models of Sinsheimer et al. [2003] (data not shown). On the other hand, when the true model involves interaction parameters (Supplementary Figure 25), this does not generally appear to generate an apparent imprinting effect (apart from when using logistic regression) but the estimates of *R*_1_, *R*_2_, and *S*_1_ are again altered.

### DIFFERENT CHOICES OF PARAMETERIZATION

To examine the relationship between our parameterization and those previously proposed, and to illustrate some of the resulting difficulties in interpretation, we analyzed a single simulated example data set under a variety of different models. The data set consisted of 4,718 individuals comprising 500 case/parent trios together with 200 of each of the following units: case/mother duos, case/father duos, cases, mothers of cases, (both) parents of cases, (both) parents of controls, control/mother duos, control/fathers duos, controls, plus an additional 218 fathers of cases. (All of these can be used as input to EMIM, see Discussion). This relatively large sample size was chosen to make it easier to compare or distinguish between different models, rather than being intended to be especially realistic. Data were simulated assuming all effects (child's genotype, mother's genotype, interactions and imprinting) operated, although details of the simulation model used are not particularly relevant, as we focus here simply on comparing the different models.

Table VII shows the results. All models were fitted in EMIM. All models include mother and child genotype effects, but differ with respect to what other parameters are included and the parameterization used. For all models, the null log likelihood (with all parameters set to 1) was identical, as expected. The differences between null and alternative log likelihoods are much larger than would generally be expected in complex disease studies (leading to very small *p* values for rejecting the null hypothesis of no effects) on account of the large sample size and relatively strong effects (relative risks) assumed. These results are not intended to be particularly realistic but serve as a useful illustrative example for demonstrating equivalences between various models.

Rows 1–4 show the parameter estimates and maximized log likelihoods for models that include a single imprinting effect in addition to mother and child genotype effects. The maximized log likelihoods are seen to be identical, regardless of what parameterization is used for the imprinting effect, however the parameter estimates for the imprinting effect and for *R*_1_ and *R*_2_ vary. Using the original Weinberg et al. [1998] parameterization (rows 1 and 2), the effects (*R*_1_, *R*_2_, *I*_m_) when imprinting is modeled as maternal effect (row 1) may be written in terms of the effects (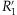, 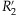, 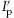) when imprinting is modeled as paternal effect (row 2) as follows: 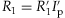, 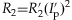, 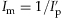. These relationships are as expected from Table I. Using the later Weinberg [1999b] parameterization (rows 3 and 4), we find the parameters to be identical to those in the earlier parameterization, except for *R*_2_ which may be written as 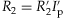. Although the fit of all four models is the same, and thus each provides identical inference concerning the presence (or absence) of an imprinting effect, the interpretation of the parameter estimates, particularly with respect to the magnitude of child's own genotype effects, is clearly very different.

Row 5 of Table VII shows the results from the Sinsheimer et al. [2003] MFG test (parameterization 1A), which includes a single incompatibility parameter γ_01_. For these data, this model appears to fit better than the models that include a single imprinting effect, although we have found in other simulations (data not shown) that an imprinting effect can often masquarade as an incompatibility effect, and vice versa, suggesting that it may be difficult to distinguish between these mechanisms in practice. If we model interaction via the EMIM parameterization using a single parameter γ_11_ (row 6), we get a slightly different (and, for these data, slightly better fitting) likelihood. Interestingly, this model appears to provide equivalent inference to the model (row 7) of Palmer et al. [2006] applied to SNPs (although note that the model used by Palmer et al. [2006] was originally designed for use with the multiallelic *HLA* system, and so may have different properties in that application). If we model interaction via the EMIM parameterization using a single parameter γ_22_ (row 8), we get a different (and, for these data, less well-fitting) likelihood.

The MFG test (parameterization 1B) shown in row 9 includes two incompatibility parameters (γ_01_ and γ_21_) and gives a better fit to these data than any of the single-interaction models. An identical fit is obtained by our default EMIM interaction (γ_11_ and γ_22_) parameterization (row 10), as expected from our previous discussion of these two parameterizations (see Methods). These models thus provide identical inference concerning the presence (or absence) of interactions, however the parameter estimates for the interactions and main genotype effects vary, as the models vary with respect to which genotype categories should be considered as “baseline” and whether the interaction term is modeled as a “compatibility” or “incompatibility” effect.

Rows 11–13 of Table VII show three models that appear to provide equivalent inference: the 6 df model used by Parimi et al. [2008] and Li et al. [2009], the model of Palmer et al. [2006] with the addition of an imprinting (*I*_p_) parameter, and the 6 df EMIM model with *I*_p_ and γ_11_ included. All of these models include a single imprinting parameter and a single-interaction parameter, and fit almost as well as the EMIM saturated 7 df model (rows 16 and 17), which may explain the high power found by Parimi et al. [2008] for this parameterization. (Note that this 6 df model would be saturated when modeling data purely from case/mother vs. control/mother duos, but case/parent trios allow the estimation of seven parameters of interest.) Alternative 6 df models shown in rows 14 and 15 (EMIM with *I*_m_ and γ_22_ included, or the MFG test (parameterization 1A) with *I*_p_ added) appear to fit rather less well.

Rows 16 and 17 illustrate the fact that a fully saturated model (containing two interactions and one imprinting effect) can be obtained either through the default EMIM parameterization or through the addition of an imprinting effect to the MFG test of Sinsheimer et al. [2003] (parameterization 1B). We found that identical results (in terms of model fit) could be obtained regardless of whether imprinting was modeled as a term *I*_m_ or *I*_p_, however only the MFG test (parameterization 1B) allowed the imprinting effect to be identifiable when modeled via the later Weinberg [1999b] parameterization (in which case, an identical fit was obtained). Although our default EMIM parameterization is based on the usual statistical approach to modeling interactions, for biological interpretability the MFG parameterization would seem more intuitive, as well as having this advantage of allowing imprinting to be modeled via either the original [Weinberg et al., 1998] or later [Weinberg, 1999b] parameterization. We have seen in this example that, where identifiable, the different imprinting parameterizations generally provide equivalent inference, but the different interaction parameterizations do not necessarily provide equivalent inference, unless both interaction terms are included. To distinguish between interaction and imprinting effects, both types of effect would need to be included in the model.

## DISCUSSION

In this study, we have presented a method for testing and estimation of maternal effects, maternal-fetal interactions and parent-of-origin effects (imprinting), using data from either case/parent trios or case/mother duos. Our multinomial modeling approach considerably outperforms logistic regression, even when fitting models with child genotype effects alone (simulation scenario A) or maternal genotypes alone (simulation scenario B), which are straightforward to fit using logistic regression. The improvement of our method over logistic regression is even greater when fitting more complex models that include parent-of-origin effects and/or interactions. We speculate that the extra power/efficiency provided by our approach comes from the extra information that is incorporated into the modeling via Mendelian inheritance assumptions (allowing the estimation of either allele frequency or mating-type frequencies), information that is not used in logistic regression. This is consistent with the results of Chen et al. [2009] who also found that improved power could be found over standard methods by exploiting the Mendelian correlation between mother's and child's genomes.

Similar improvements in power for log-linear models (equivalent to multinomial modeling) over logistic regression have been found previously [Shi et al., 2008; Vermeulen et al., 2009; Weinberg and Shi, 2009; Weinberg and Umbach, 2005]. However, these previous investigations did not include the full range of parameters of interest (e.g. maternal-fetal genotype interactions were not considered) and power was calculated using theoretical arguments based on non-centrality parameters rather than by computer simulation, making it impossible to examine key properties of the methods such as bias, precision, and uncertainty in parameter estimation. Parimi et al. [2008] used computer simulations to compare logistic regression with log-linear models under various underlying scenarios, and also found log-linear models to give higher power, although their results were somewhat hard to interpret in view of the fact that they used different sample sizes for the different types of analysis (50 case/mother and 50 control/mother duos for logistic regression, compared to either 67 or 100 case/parent trios).

To our knowledge, ours is the first approach that allows the estimation of these kinds of complex effects using data from case/mother duos alone (without the incorporation of fathers or control samples of various types), although we note that the performance of our approach is considerably improved by the incorporation of either fathers or various types of control sample. In particular, case/parent trios provide substantially higher power and better parameter estimation than is provided by case/mother duos, suggesting that the collection of fathers, where possible, can add considerable value to a study. Although in our simulations we considered the two extreme cases of *no* fathers being available (the data set consisting solely of case/mother duos) or *all* fathers available (the data set consisting solely of case/parent trios), in practice one may combine these data structures by multiplying together the relevant likelihood contributions. In a similar fashion one may also include other data structures such as case/parent trios where the mother is missing (i.e. case/father duos), where the child is missing (i.e. parents of cases) or where only one parent is available (i.e. mothers or fathers of cases), simply by collapsing the rows of Table II appropriately and fitting the resulting multinomial likelihood. Our approach thus provides a general framework for analysis of case/parent trios even when one (or more) individuals within a trio are missing (via direct maximization of the product of multinomial likelihoods rather than by using an EM algorithm), allowing one to maximize information from all available data.

In our computer simulations, we assumed a relatively common disease (baseline penetrance α = 0.1) and disease allele frequency (*A*_2_ = 0.3) in order to reduce the computational time required to generate sufficient simulation replicates. We therefore simulated population-based control samples rather than unaffected controls. This is an important distinction as the control samples are used to help estimate the allele frequencies or mating-type frequencies in the population; for a common disease, these frequencies will be distorted if estimated conditional on (unaffected) disease status in the child [Weinberg and Shi, 2009]. We found similar results to those presented here when we varied the disease allele frequency and/or simulated a rare disease together with known unaffected controls (data not shown), indicating that, for a rare disease, either unaffected or population-based (of unknown disease status) controls can be used.

One appealing property of family-based designs is the robustness they often provide to population stratification. We found only one method (Method 2b, applied to case/parent trios) that provided complete robustness to population stratification. This is not unexpected since population stratification will generally induce departure from HWE, meaning that methods that make a HWE and random mating assumption are likely to be compromised when these assumptions do not hold. Methods that make use of additional control samples (even without assuming HWE and random mating) also suffer in the presence of population stratification on account of the fact that the underlying mating-type frequencies and thus parameters µ_1_−µ_6_ will differ between parents of cases and parents of controls. In case/control studies, alternative methods have been developed to deal with population stratification [Devlin and Roeder, 1999; Price et al., 2006; Pritchard et al., 2000]. However, incorporation of covariates (for example principal component scores from an eigenvector analysis [Price et al., 2006] as is often carried out in case/control studies [WTCCC, 2007]) is problematic in a multinomial or log-linear modeling framework, and so it is unclear whether this approach would be feasible here, in preference to simply sampling from an ethnically homogenous population.

Method 2b, applied to case/parent trios, is formally equivalent to the original log-linear model approach of Weinberg et al. [1998] and Weinberg [1999a,b] and is closely related to the case/pseudocontrol approach proposed by Cordell and Clayton [2002] and Cordell et al. [2004] [based on previous work by Schaid, 1996; Schaid and Sommer, 1993; Self et al., 1991]. The case/pseudocontrol approach loses some efficiency by conditioning both on parental genotypes and sufficient statistics for parameters of interest (such as parent-of-origin effects) [Cordell et al., 2004]. However, the approach does have the advantage that comes with being embedded in a (conditional logistic) regression framework of allowing the incorporation of covariates (such as other genetic or environmental factors) as well as factors such as gene-gene and gene-environment interactions. Thus, the case/pseudocontrol approach may be worth considering if inclusion of covariates (including those related to adjusting for population stratification) is an important consideration in a given study.

Our method has been implemented in a freely available software package, EMIM. EMIM is a Fortran program that has been most extensively tested under Linux, although in theory should work on any operating system (e.g. Microsoft Windows, Apple Macintosh) that has a Fortran compiler available. EMIM makes use of a subroutine MAXFUN, originally written as part of the S.A.G.E. [1994] package. EMIM allows the user to specify several different input files containing data from different types of structures (e.g. case/parent trios, case/mother duos, parents of controls, etc.) and also allows the user to specify which parameters to estimate or fix, and what parameter restrictions or modeling assumptions (e.g. Hardy-Weinberg and random mating) are to be used. Competing software that can be used to fit similar models includes a set of SAS macros available from the Weinberg website (http://www.niehs.nih.gov/research/atniehs/labs/bb/staff/weinberg/index.cfm). These macros fit the specific models described in Weinberg [1999a] and Weinberg and Umbach [2005] but could presumably be adapted by an experienced SAS user to implement alternative models and parameter restrictions. R code is available for implementing the approaches of Li et al. [2009] and Chen et al. [2009]. The MFG test of Sinsheimer et al. [2003] has been implemented in the software package Mendel [Lange et al., 2001, 2005]. We have also included functionality for fitting the parameters of the MFG test and the tests of Li et al. [2009] and Palmer et al. [2006] (applied to diallelic systems) in our package EMIM. Models that do not include consideration of missing parents or imprinting effects (such as the MFG test) could also be fit in standard statistical software for log-linear modeling but, as discussed previously, missing data (e.g. with respect to the father's genotype or the parental origin of the child's alleles) create problems associated with the fact that certain cell probabilities will correspond to sums of products (rather than purely products) of the parameters of interest, making these models difficult to fit in practice.

Probably, the most convenient software package that has been used previously [Shi et al., 2008; Vermeulen et al., 2009; Weinberg and Shi, 2009] for fitting these kinds of model is the program LEM [van Den Oord and Vermunt, 2000]. LEM allows fitting of log-linear models via an expectation maximization algorithm. LEM scripts for fitting various models are available from the Weinberg website (http://www.niehs.nih.gov/research/atniehs/labs/bb/staff/weinberg/index.cfm); again the experienced user could presumably use these as a basis for fitting alternative models. We found LEM to give identical results to our program EMIM when applied to the same data sets using the same parameter restrictions (data not shown). Since LEM is a Microsoft Windows “point and click” type program, it is not especially convenient for performing many repeated analyses (e.g. when performing a computer simulation with a large number of replicates, or when applying the method to large numbers of SNPs such as are generated in a genome-wide association study). However, in theory one could invoke LEM through the MS-DOS prompt, which would allow more convenient scripting capabilities when performing large numbers of repeated analyses.

Although the methodology (and software) for investigating effects of imprinting, maternal genotype, and maternal-fetal genotype interactions is now available, interpretation of the parameter estimates obtained can be quite complex, owing to collinearities and lack of identifiability between various sets of parameters. In particular, the parameterization used for imprinting and interaction effects requires some care, as this can affect the interpretation of other estimated parameters. For modeling interactions, the MFG parameterization of Sinsheimer et al. [2003] or the more restricted models used by Palmer et al. [2006], Parimi et al. [2008] and Li et al. [2009] would seem most biologically intuitive, although we note that all of these models are essentially captured via our default (statistically based) EMIM parameterization. Such complexities in parameter definition and interpretation suggest that this kind of modeling should perhaps be best considered as a first step toward disentangling such complex effects, in order to generate more focused hypotheses that may be further investigated experimentally.

## WEB RESOURCES

Software implementing the proposed approach (the EMIM program) will be made available on publication from our website: http://www.staff.ncl.ac.uk/heather.cordell/software.html

## APPENDIX A: DERIVATION OF APPARENT GENOTYPE RELATIVE RISKS

Here, we use Table II to derive formulae for the apparent genotype relative risks in cases vs. controls, or in mothers of cases vs. mothers of controls. The genotype relative risks for cases vs. (population) controls can be derived from the penetrances *P*(dis|*g*_c_) where “dis” indicates the event that a child is affected with disease and *g*_c_ denotes the child's genotype (22, 12, 21 or 11). Let *g*_m_ and *g*_f_ denote the unordered genotypes in the mother and father, while for now we consider ordered (maternal/paternal) genotypes for the child (i.e. we distinguish between *g*_c_ = 12 and *g*_c_ = 21). The penetrances may be written as:

Now  and  are given in columns 4 and 5 of Table II. Assuming random mating and Hardy-Weinberg equilibrium (HWE), *P*(*g*_m_, *g*_f_) is given in column 6 of Table II and *P*(*g*_c_) can be calculated similarly as , *A*_1_*A*_2_, *A*_2_*A*_1_, and  for genotypes *g*_c_ = 11, 12, 21, 22, respectively. Thus, by multiplying together the appropriate columns (4, 5, and 6) of Table II, dividing by *g*_c_ and summing the resulting 16 quantities, we obtain:

Thus, the relative risks *RR*_12_ and *RR*_22_ for the unordered case genotypes 12 and 22 (relative to genotype 11) may be calculated as  and .

A similar approach may be used to derive formulae for the apparent genotype relative risks in mothers of cases vs. mothers of controls. The penetrances for mothers correspond to *P*(mother has diseased child|*g*_m_), where *g*_m_ denotes the mothers (unordered) genotype (22, 12, or 11). This may be written as:

Again by multiplying together the columns 4, 5, and 6 of Table II, dividing by *g*_m_ (which takes values , 2*A*_1_A_2_, and  for genotypes *g*_m_ = 11,12, and 22, respectively) and summing the resulting 16 quantities, we obtain:

Thus, the relative risks *RR*_12_ and *RR*_22_ for the unordered mother-of-case genotypes 12 and 22 (relative to genotype 11) may be calculated as *RR*_12_ = *P*(dis|*g*_m_ = 12)/*P*(dis|*g*_m_ = 11) and *RR*_22_ = *P*(dis|*g*_m_ = 22)/*P*(dis|*g*_m_ = 11). Formulae for these relative risks, together with those previously calculated for the cases, are shown in Table VI.

## APPENDIX B: FORMULAE FOR *K* AND Σ

So

If HWE and random mating are to be assumed, the terms  in the above equations may be replaced by functions of the allele frequency *A*_2_ (and *A*_1_ = 1−*A*_2_) as follows:
