# Supplementary material for: Investigation of Maternal Effects, Maternal-Fetal Interactions and Parent-of-Origin Effects (Imprinting), Using Mothers and Their Offspring
Source: Genet Epidemiol. 2011 Jan;35(1):19–45. doi: 10.1002/gepi.20547 (PMC3025173; doi:10.1002/gepi.20547)
Supplement: Supplementary file 1 [file gepi0035-0019-SD1.doc]

Supplementary Table I: Multinomial probabilities for genotypes of controls

| Genotype | Cell probability | Cell probability assuming HWE |
| --- | --- | --- |
| 22 | *μ*_1_+2*μ*_2_+*μ*_4_ | *A*_2_^2^ |
| 12 | 2*μ*_2_+2*μ*_3_+2*μ*_4_+2*μ*_5_ | 2*A*_2_*A*_1_ |
| 11 | *μ*_4_+2*μ*_5_+*μ*_6_ | *A*_1_^2^ |
