# Supplementary material for: Investigation of Maternal Effects, Maternal-Fetal Interactions and Parent-of-Origin Effects (Imprinting), Using Mothers and Their Offspring
Source: Genet Epidemiol. 2011 Jan;35(1):19–45. doi: 10.1002/gepi.20547 (PMC3025173; doi:10.1002/gepi.20547)
Supplement: Supplementary file 3 [file gepi0035-0019-SD3.doc]

Supplementary Table III: Multinomial cell probabilities for genotype combinations in control/mother duos

| Cell (row)  index | Genotypes^a^  g_m_ g_c_ | P(g_m_, g_c_) |
| --- | --- | --- |
| 1 | 22 22 | *μ*_1_*+μ*_2_ |
| 2 | 22 12 | *μ*_2_*+μ*_3_ |
| 3 | 12 22 | *μ*_2_*+μ*_4_ |
| 4 | 12 12 | *2μ*_4_*+μ*_5_*+μ*_2_ |
| 5 | 12 11 | *μ*_4_*+μ*_5_ |
| 6 | 11 12 | *μ*_3_*+μ*_5_ |
| 7 | 11 11 | *μ*_5_*+μ*_6_ |

^a^ g_m_ refers to the unordered alleles in the mother. g_c_ refers to the unordered alleles in the child
