# Supplementary material for: Investigation of Maternal Effects, Maternal-Fetal Interactions and Parent-of-Origin Effects (Imprinting), Using Mothers and Their Offspring
Source: Genet Epidemiol. 2011 Jan;35(1):19–45. doi: 10.1002/gepi.20547 (PMC3025173; doi:10.1002/gepi.20547)
Supplement: Supplementary file 4 [file gepi0035-0019-SD4.doc]

Supplementary Table IV: Genotype relative risk estimates when analysing either the child (cases versus controls), or the mother (mothers of cases versus mothers of controls) for different sets of parameter values (i.e. different underlying genetic models). Parameters are as defined in the text

| True parameter values | | | | | | | | | | | Apparent genotype relative risks | | | | Predicted power^a^ for P= 0.01 when analysing 500 cases/controls | |
| --- | --- | --- | --- | --- | --- | --- | --- | --- | --- | --- | --- | --- | --- | --- | --- | --- |
|  |  |  |  |  |  |  |  |  |  |  | Child | | Mother | | Child | Mother |
| *A*_2_ | *R*_1_ | *R*_2_ | *S*_1_ | *S*_1_ | *I_m_* | *I_p_* | * γ*_11_ | * γ*_12_ | * γ*_21_ | * γ*_22_ | RR_12_ | RR_22_ | RR_12_ | RR_12_ |  |  |
| 0.2 | 1 | 2 | - | - | - | - | - | - | - | - | 1 | 2 | 1.1 | 1.2 | 0.18 | 0.04 |
| 0.2 | 1 | 4 | - | - | - | - | - | - | - | - | 1 | 4 | 1.3 | 1.6 | 0.97 | 0.39 |
| 0.2 | 2 | 2 | - | - | - | - | - | - | - | - | 2 | 2 | 1.33 | 1.67 | 0.99 | 0.48 |
| 0.2 | 4 | 4 | - | - | - | - | - | - | - | - | 4 | 4 | 1.75 | 2.5 | 1.00 | 0.99 |
| 0.2 | 2 | 4 | - | - | - | - | - | - | - | - | 2 | 4 | 1.5 | 2 | 1.00 | 0.83 |
| 0.2 | - | - | 1 | 2 | - | - | - | - | - | - | 1.1 | 1.2 | 1 | 2 | 0.04 | 0.18 |
| 0.2 | - | - | 1 | 4 | - | - | - | - | - | - | 1.3 | 1.6 | 1 | 4 | 0.39 | 0.97 |
| 0.2 | - | - | 2 | 2 | - | - | - | - | - | - | 1.33 | 1.67 | 2 | 2 | 0.48 | 0.99 |
| 0.2 | - | - | 4 | 4 | - | - | - | - | - | - | 1.75 | 2.5 | 4 | 4 | 0.99 | 1.00 |
| 0.2 | - | - | 2 | 4 | - | - | - | - | - | - | 1.5 | 2 | 2 | 4 | 0.83 | 1.00 |
| 0.2 | - | - | - | - | 2 | - | - | - | - | - | 1.5 | 2 | 1.5 | 2 | 0.83 | 0.83 |
| 0.2 | - | - | - | - | 3 | - | - | - | - | - | 2 | 3 | 2 | 3 | 0.99 | 0.99 |
| 0.2 | - | - | - | - | - | 2 | - | - | - | - | 1.5 | 2 | 1 | 1 | 0.83 | 0.01 |
| 0.2 | - | - | - | - | - | 3 | - | - | - | - | 2 | 3 | 1 | 1 | 0.99 | 0.01 |
| 0.2 | - | - | - | - | - | - | 0.5 | - | - | 0.5 | 0.75 | 0.9 | 0.75 | 0.9 | - | - |
| 0.2 | - | - | - | - | - | - | 2 | - | - | 2 | 1.5 | 1.2 | 1.5 | 1.2 | 0.47 | 0.47 |
| 0.2 | - | - | - | - | - | - | 0.5 | 2 | 2 | 0.5 | 0.85 | 1.7 | 0.85 | 1.7 | - | - |
| 0.1 | 1 | 2 | - | - | - | - | - | - | - | - | 1 | 2 | 1.05 | 1.1 | 0.03 | 0.01 |
| 0.1 | 1 | 4 | - | - | - | - | - | - | - | - | 1 | 4 | 1.15 | 1.3 | 0.23 | 0.05 |
| 0.1 | 2 | 2 | - | - | - | - | - | - | - | - | 2 | 2 | 1.41 | 1.82 | 0.97 | 0.42 |
| 0.1 | 4 | 4 | - | - | - | - | - | - | - | - | 4 | 4 | 2.04 | 3.08 | 1.00 | 0.99 |
| 0.1 | 2 | 4 | - | - | - | - | - | - | - | - | 2 | 4 | 1.5 | 2 | 0.99 | 0.59 |
| 0.1 | - | - | 1 | 2 | - | - | - | - | - | - | 1.05 | 1.1 | 1 | 2 | 0.01 | 0.03 |
| 0.1 | - | - | 1 | 4 | - | - | - | - | - | - | 1.15 | 1.3 | 1 | 4 | 0.05 | 0.23 |
| 0.1 | - | - | 2 | 2 | - | - | - | - | - | - | 1.41 | 1.82 | 2 | 2 | 0.42 | 0.97 |
| 0.1 | - | - | 4 | 4 | - | - | - | - | - | - | 2.04 | 3.08 | 4 | 4 | 0.99 | 1.00 |
| 0.1 | - | - | 2 | 4 | - | - | - | - | - | - | 1.5 | 2 | 2 | 4 | 0.59 | 0.99 |
| 0.1 | - | - | - | - | 2 | - | - | - | - | - | 1.5 | 2 | 1.5 | 2 | 0.59 | 0.59 |
| 0.1 | - | - | - | - | 3 | - | - | - | - | - | 2 | 3 | 2 | 3 | 0.98 | 0.98 |
| 0.1 | - | - | - | - | - | 2 | - | - | - | - | 1.5 | 2 | 1 | 1 | 0.59 | 0.01 |
| 0.1 | - | - | - | - | - | 3 | - | - | - | - | 2 | 3 | 1 | 1 | 0.98 | 0.01 |
| 0.1 | - | - | - | - | - | - | 0.5 | - | - | 0.5 | 0.75 | 0.95 | 0.75 | 0.95 | - | - |
| 0.1 | - | - | - | - | - | - | 2 | - | - | 2 | 1.5 | 1.1 | 1.5 | 1.1 | 0.41 | 0.41 |
| 0.1 | - | - | - | - | - | - | 0.5 | 2 | 2 | 0.5 | 0.8 | 1.85 | 0.8 | 1.85 | - | - |
| 0.3 | 1.5 | 2.25 | - | - | - | - | - | - | - | - | 1.5 | 2.25 | 1.25 | 1.5 | 0.96 | 0.35 |
| 0.3 | - | - | 1.5 | 2.25 | - | - | - | - | - | - | 1.25 | 1.5 | 1.5 | 2.25 | 0.35 | 0.96 |
| 0.3 | 1.5 | 2.25 | - | - | 1.8 | - | - | - | - | - | 2.1 | 4.05 | 1.85 | 2.7 | 1.00 | 0.99 |
| 0.3 | 1.5 | 2.25 | - | - | - | 1.8 | - | - | - | - | 2.1 | 4.05 | 1.25 | 1.5 | 1.00 | 0.35 |

^a^ Power calculated using the Genetic Power Calculator (<http://pngu.mgh.harvard.edu/~purcell/gpc/cc2.html>) This program does not allow the calculation to be performed for relative risks less than 1, so powers for parameter values in this category are not calculated (and are denoted “-”).
